# Neuropsychological Measures that Predict Progression from Mild Cognitive Impairment to Alzheimer's type dementia in Older Adults: a Systematic Review and Meta-Analysis

**DOI:** 10.1007/s11065-017-9361-5

**Published:** 2017-10-10

**Authors:** Sylvie Belleville, Céline Fouquet, Carol Hudon, Hervé Tchala Vignon Zomahoun, Jordie Croteau, Consortium for the Early Identification of Alzheimer’s disease-Quebec

**Affiliations:** 1 Research Center of the Institut Universitaire de Gériatrie de Montréal, 4565 Chemin Queen Mary, Montréal, Québec, H3W 1W5 Canada; 2 Université de Montréal, CP 6128 Succ. Centre Ville, Montréal, Québec, H3C-1J7 Canada; 3 Université Laval, Pavillon Félix-Antoine-Savard, 2325, rue des Bibliothèques, Local 1546, Québec, Québec G1V 0A6 Canada; 4 CERVO Brain Research Center, 2601, de la Canardiere, Québec, Québec G1J 2G3 Canada; 5 Health and Social Services Systems, Knowledge Translation and Implementation component of the Quebec SPOR-SUPPORT Unit, Université Laval, Québec, Québec G1L 2E8 Canada; 6 Population Health and Practice-Changing Research Group, Research Centre of CHU de Québec- Université Laval, Québec, Québec G1L 2E8 Canada

**Keywords:** Alzheimer’s disease, Mild cognitive impairment, Neuropsychology, Diagnosis, Cognitive tests, Predictive accuracy

## Abstract

This study aimed to determine the extent to which cognitive measures can predict progression from mild cognitive impairment (MCI) to Alzheimer’s type dementia (AD), assess the predictive accuracy of different cognitive domain categories, and determine whether accuracy varies as a function of age and length of follow-up. We systematically reviewed and meta-analyzed data from longitudinal studies reporting sensitivity and specificity values for neuropsychological tests to identify individuals with MCI who will develop AD. We searched articles in Medline, Cochrane, EMBASE, PsycINFO, and the Web of Science. Methodological quality was assessed using the STARDem and QUADAS standards. Twenty-eight studies met the eligibility criteria (2365 participants) and reported predictive values from 61 neuropsychological tests with a 31-month mean follow-up. Values were pooled to provide combined accuracy for 14 cognitive domains. Many domains showed very good predictive accuracy with high sensitivity and specificity values (≥ 0.7). Verbal memory measures and many language tests yielded very high predictive accuracy. Other domains (e.g., executive functions, visual memory) showed better specificity than sensitivity. Predictive accuracy was highest when combining memory measures with a small set of other domains or when relying on broad cognitive batteries. Cognitive tests are excellent at predicting MCI individuals who will progress to dementia and should be a critical component of any toolkit intended to identify AD at the pre-dementia stage. Some tasks are remarkable as early indicators, whereas others might be used to suggest imminent progression.

**Electronic supplementary material:**

The online version of this article (10.1007/s11065-017-9361-5) contains supplementary material, which is available to authorized users.

## Introduction

Alzheimer’s disease (AD) is progressive. It is only diagnosed with certainty when the post-mortem neuropathological examination reveals the presence of amyloid plaques and neurofibrillary tangles in the brains of patients who have suffered from the clinical symptoms prior to their death. Antero-mortem diagnosis thus relies on a set of clinical inclusion and exclusion criteria (e.g., APA 2013; McKhann et al. 2011). Based on current criteria, the diagnosis of probable AD is given when patients have dementia which is defined as cognitive impairment that involves at least two cognitive domains and is sufficiently severe that it interferes with activities of daily living (APA 2013; McKhann et al. 2011). However, dementia corresponds to the end phase of AD. Studies on patients suffering from the autosomal dominant version of the disease and population studies that have followed patients or analyzed their performance retrospectively, suggest that the disease probably starts 10–15 years earlier than when patients typically receive their diagnosis (Amieva et al. 2008; Bateman et al. 2012). This late diagnosis considerably impedes research as it limits the ability to identify the early mechanisms that trigger the disease and contribute to its progression. Furthermore, a considerable effort has been devoted to finding a curative treatment and identifying effective lifestyle prevention strategies for AD. When discovered, such treatment or preventative approaches will have to be provided before the disease has produced major damage to the brain to have the most benefit. These issues have motivated a substantial research effort towards detection at an earlier stage of the disease, prior to dementia.

Mild cognitive impairment (MCI) has been of particular interest in this regard. Individuals with MCI complain about their memory and show mild cognitive deficits when tested with objective cognitive measures (Petersen 2000; Gauthier et al. 2011; Albert et al. 2011), but their cognitive impairment is not sufficiently severe to meet the criteria for dementia. Yet, these individuals have a ten-fold greater risk of progression to dementia than the general population, suggesting that many are actually in a pre-dementia phase of the disease (Gauthier et al. 2011; Petersen 2000). The cognitive assessment performed by neuropsychologists is a central component for the diagnosis of AD at the dementia phase, and it is thus critical to have access to sensitive and specific cognitive measures. Relying on appropriate measures is even more critical during the MCI phase, in which cognitive impairment is mild and functional impact minimal. Differentiating the cognitive deficits related to incipient AD from those related to cognitive fluctuations due to normal aging or other non-morbid conditions is particularly challenging, because impairment is mild at this phase.

Many studies have characterized the cognition of people with MCI relative to that of healthy older adults using cross-sectional designs (for reviews, see Belleville et al. 2008; Belleville et al. 2014a). These studies have provided clinicians with cognitive characterization of this early phase and clinical tools that can be used to determine whether older adults meet the criteria for MCI. However, not all individuals who meet the criteria for MCI will progress to dementia and one major challenge is to identify tests that differentiate between individuals with MCI who will remain stable and those who will progress toward dementia. Many studies have attempted to identify early markers of future progression. These studies rely on a longitudinal design in which MCI participants are tested at entry on a set of cognitive measures and followed over time to identify progressors versus non-progressors. Regression analyses can then be used to assess whether performance on cognitive tests completed at entry indeed predict clinical progression.

Many of these prognostic studies relied on comparable tests and it is therefore possible to pool the data to perform quantitative meta-analyses. This allows us to address many important questions related to early cognitive diagnosis. One major question is whether cognitive tests fare well in predicting progression from MCI to dementia, which is crucial in a context where much effort is devoted to developing biomarkers of progression. Cognitive assessments are widely available and relatively low-cost. Thus, it is important to determine their predictive accuracy and provide quantitative values so that they can be compared to more costly biomarkers. Previous reviews have indicated that the predictive accuracy reported in the literature is extremely variable (Belleville et al. 2014a). This could be due to a wide range of factors, including the cognitive domain measured by the task, length of follow-up, or the characteristics of the patient. Tests might also differ in terms of their specificity and sensitivity. In prognostic studies, sensitivity refers to the correct identification of progressors, whereas specificity refers to the correct rejection of non-progressors.

One important question is to what extent different domains determine overall predictive accuracy, sensitivity, and specificity. For example, some of the most well-accepted clinical criteria have proposed that cued recall (Dubois et al. 2007) or delayed memory (Albert et al. 2011) may be the most highly affected in MCI. It is thus important to assess whether this is the case by conducting an appropriate meta-analysis of the data. Another issue is the role of non-memory domains in early diagnosis. Some studies have found that working memory, executive functions, and language can be impaired in MCI (Belleville et al. 2007; Joubert et al. 2008; Saunders and Summers 2010). However, these studies were based on cross-sectional group comparisons and it is unclear whether these tests are sufficiently sensitive to predict dementia at the individual level. In contrast, non-memory-based tests may have value for specificity, because impairment of non-memory aspects may be more problematic when present. A few studies have examined multiple tests or those that cover a wide range of cognitive functions as predictors of dementia. This strategy may optimize accuracy by pooling tests with a different balance between their sensitivity and specificity (Summers and Saunders 2012; Belleville et al. 2014b).

A related question is the impact that age might have on predictive values of different tests. Age is a potentially important factor because aging itself is accompanied by a specific set of cognitive deficits and because mixed dementia is more frequent in older patients. Thus, older age might be associated with a larger contribution from non-memory domains, particularly executive functions. The length of follow-up is a major issue as well. Studies with shorter follow-ups run the risk of identifying an individual as stable even though they would have progressed to dementia had there been a longer follow-up. Thus, shorter follow-ups increase the likelihood of false negatives and might reduce test sensitivity for the task being evaluated, whereas a longer follow-up should increase sensitivity. A sufficiently long follow-up can be used as a proxy for how far the patient was from the diagnosis when the test was given. Thus, assessing the impact of follow-up length is also informative for identifying whether a task is more useful as a very early marker or as a reflection of imminent progression. A task found to be sensitive at a longer follow-up might be particularly well suited as an early indicator of future dementia. Thus, studies with longer average follow-ups might identify earlier predictors. In turn, a task with a relatively low sensitivity at longer follow-ups but with an excellent value at shorter ones would reflect imminent progression.

Our goal was to conduct a systematic review and meta-analysis to determine the extent to which cognitive measures predict progression from MCI to dementia of the AD type. We used data from longitudinal studies reporting sensitivity and specificity values for cognitive tests to identify individuals with MCI who will progress to a diagnosis of AD in the future. The present study does not replicate or update existing reviews. Other authors have systematically reviewed the diagnostic performance of cognitive tests to cross-sectionally identify people with MCI (Ozer et al. 2016) or dementia (Tsoi et al. 2015), and one study has examined which screening tools best predict progression to dementia in primary care patients (Lischka et al. 2012). However, no review has yet investigated the sensitivity and specificity of a variety of cognitive tests to predict progression to AD among individuals with MCI.

The specific questions addressed in this meta-analysis and systematic review are 1) the predictive value of cognitive measures to predict future progression from MCI to dementia and more specifically, their global (or average) sensitivity (correct identification of progressors) and specificity (correct rejection of non-progressors), 2) the impact of age on predictive accuracy, 3) the average length of follow-up from reported studies and its impact on predictive accuracy, and 4) the benefits of combining different cognitive domains.

## Materials and Methods

This systematic review was reported in accordance with the PRISMA statement (Liberati et al. 2009). The protocol was not registered, but it was predetermined, and PICOS statements were used to identify the studies to be included in the review and meta-analyses.

### Eligibility Criteria

Criteria for including or excluding articles were determined a priori. The PICOS approach was adapted to formulate the research question. Studies were included if they were longitudinal and designed to assess the sensitivity and specificity of neuropsychological tests to determine future progression from mild cognitive deficit to dementia of the Alzheimer type. Operational inclusion criteria were the following i) articles written in English or French, ii) studies using a prospective or retrospective longitudinal design, iii) at baseline, participants were identified as older adults with MCI and no dementia or a related terminology, iv) neuropsychological or cognitive tests were used as predictors and were sufficiently well described to allow replication, v) participants had a follow-up of at least 1 year, vi) the outcome was a diagnosis of dementia of the Alzheimer disease’s type based on the DSM-III-R criteria (APA 1987), DSM-IV criteria (APA 1994) or National Institute of Neurological and Communication Disorders and Stroke/Alzheimer’s Disease and Related Disorders Association (NINCDS-ADRDA) criteria (McKhann et al. 1984), and supported by a medical examination or a medical consensus panel, and vii) absolute values for false positives, false negatives, true positives, and true negatives were reported or 2x2 tables could be constructed based on reported data. No publication date restriction was imposed.

There were also several exclusion criteria. Studies were excluded i) if the diagnosis of AD was based on a single neuropsychological test or if there was no definition of the criteria used for the diagnosis of AD, ii) if the study targeted populations with other neurological diseases or neurodevelopmental disorders (e.g., HIV associated dementia, Parkinson’s disease, Down Syndrome), iii) if it used a cross-sectional design and iv) if there were insufficient methodological details to allow replication. Furthermore, only the more recent data were included if the same population sample was used in more than one study.

### Information Sources and Search

A computer-based search was performed from five electronic databases: MEDLINE using PubMed (1946 - Present), Embase using OVID (1947 - Present), Cochrane (1918 - Present), PsycINFO (1967 - Present), and Web of Science. The last search was carried out on January 29th, 2014. In addition, the reference lists from articles of interest were searched for additional references. The full electronic search strategy for MEDLINE is included in Appendix A (*Supplementary material*). The search strategy, based on the PICOS approach, was applied through a combination of five concepts that delimited our research question 1) Patient, defined as “elderly population” with “MCI”, 2) Intervention, defined as the index tests, that is, the different neuropsychological predictors, 3) Comparison, defined by the type of analysis expected in the selected studies i.e.*,* those including predictive values for the neuropsychological predictors, 4) Outcome, defined as the predicted outcome which was “Alzheimer’s Disease” and 5) Type of study, which specified that “longitudinal studies” were the type of studies sought. The search strategy was reviewed and validated by a librarian who is an expert in database searches.

### Study Selection

The articles produced by the search strategy were first screened based on the titles and then selected by one reviewer (CF) based on the abstracts. One additional reviewer (CH or SB) independently revised the list of potential articles based on the abstracts. The full text of the articles considered to be potentially eligible was then evaluated in detail by the same reviewer for quality assessment and any unresolved issues were discussed with SB. All articles meeting eligibility criteria were included in the qualitative synthesis. Articles reporting data that could be appropriately pooled were included in the meta-analyses: this was done when at least three studies reported data for the same cognitive domain.

### Data Collection Process and Data Items

Two reviewers independently extracted data from all eligible studies using a predetermined form (standardized grid). Disagreements between the two reviewers were resolved by consensus or a third reviewer (CH or SB). The data collected from each manuscript included information on the general description of the study, participants, methodological features, results, analysis, and analytic procedure. Table S1 (*Supplementary material*) shows the items for which information was collected for all papers.

### Risk of Bias in Individual Studies

The methodological quality and the risk of bias of each study was assessed using the Quality Assessment of Diagnostic Accuracy Studies (QUADAS) tool (Whiting et al. 2003), as suggested by Cochrane guidelines (Reitsma et al. 2009). A tailored quality assessment grid was constructed to apply the quality assessment criteria proposed in QUADAS 2 (Whiting et al. 2011) adapted to prognostic studies (see Table S2 in *Supplementary material*). Study quality was assessed by a single reviewer with verification by a second reviewer for i) patient selection and population source (e.g., whether the included sample was representative of the population and whether exclusion criteria were too restrictive), ii) study design (e.g., whether it was a prospective or retrospective study and whether the investigator in charge of diagnosis at follow-up was blind to the baseline performance), iii) flow and follow-up (e.g., length of follow-up), iv) reference standard and outcome (i.e., adequate procedure for diagnosis at follow-up and absence of partial verification bias), v) index tests and prognosis (i.e., adequate description of the neuropsychological battery), and vi) analysis (e.g., whether the cut-off used for calculation of sensitivity and specificity measures was determined using independent norms). This quality assessment allowed us to classify studies as having a low (L), high (H), or low-high (LH) risk of bias.

## Analyses

Articles reporting appropriate data were included in the meta-analyses. If the prevalence was not provided, it was calculated by dividing the number of participants who had progressed at follow-up by the total number of participants at follow-up. If the number of true positives (TP), false positives (FP), true negatives (TN), and false negatives (FN) were not available, they were calculated based on the values of sensitivity and specificity using the following formula: TP = sensitivity x number of progressors, TN = specificity x number of non-progressors, FN = number of progressors minus TP, FP = number of non-progressors minus TN.

### Variables of Interest

The first aim of the meta-analyses was to obtain global estimates of predictive accuracy for various categories of neuropsychological tests. The study setting was very similar to that of diagnostic test accuracy studies, except that instead of comparing the diagnostic index test results to a gold standard, the index tests were compared to the diagnostic gold standard performed after a certain follow-up period. We therefore used the same methods as for diagnostic test accuracy meta-analyses by obtaining the global sensitivity and specificity for each test category. Sensitivity refers here to the ability of the test to identify MCI patients who will later progress to dementia or more precisely, whether progressors were impaired on the test. Specificity refers to the ability of the test to identify those who will remain stable, that is, whether stable MCI were unimpaired on the test. An ideal test should have 100% sensitivity and 100% specificity, which would result in perfect predictive accuracy. Scores higher than 0.7 are typically considered to be very good, whereas scores above 0.5 indicate good classification (Haynes et al. 2006).

Tests were grouped within 22 cognitive domain categories for systematic review (see Table 3), given the large variety of neuropsychological tests used in the various studies. The classification of tests within cognitive domain categories was performed following study selection, but prior to data analysis, by consensus between the authors and validated by the CIMA-Q cognition expert group, which comprises 12 clinicians and researchers with expertise in the neuropsychology of dementia. This led to the identification of five broad domains corresponding to cognitive components that are well-recognized and believed to depend on distinct neuroanatomical substrates: verbal episodic memory, visual episodic memory, language, executive functions and working memory, and visuo-constructive functions. An additional category was used to include brief and global measures of cognition. We then identified test categories that were judged to reveal different brain or cognitive mechanisms within each domain, or to be differentially affected by dementia. For example, we distinguished immediate from delayed recall, because delayed recall is generally believed to reflect the consolidation processes that occurs within the hippocampus to a larger extent than immediate recall. We distinguished studies using oriented encoding, where the examiner provides cues during the encoding phase, as well as studies using cued recall, where the examiner provides cues during the retrieval phase, because some have argued that providing cues at encoding or retrieval reduces the contribution of executive attention to memory performance.

We separately analyzed studies that identified a combination of tests predicting progression in the systematic review, but they were not included in the meta-analysis. Each cognitive domain category was meta-analyzed separately. The minimum number of studies required to perform a diagnostic accuracy meta-analysis is three (HIQA – IRELAND, IQWiG – GERMANY 2015). The 14 domains that met this criterion were the following 1) among verbal memory tests, verbal immediate recall, paragraph delayed recall, word-list free delayed recall with non-oriented encoding, word-list free delayed recall with oriented encoding, word-list cued delayed recall with oriented encoding, and word-list recognition 2) among visual memory tests, delayed recall 3) among language tests, naming tests, tests of semantic knowledge, and semantic fluency 4) among executive tests, switching tests and working memory tests, 5) among visuo-constructive measures, visuo-spatial tests and visuo-constructive tasks and 6) global measures.

### Meta-Analysis

The Cochrane collaboration and others (Macaskill et al. 2010; HIQA – IRELAND, IQWiG – GERMANY 2015; Dahabreh et al. 2012) strongly recommend the use of the random effects hierarchical bivariate models called *bivariate* (Reitsma et al. 2005) and *hierarchical summary receiver operating characteristic (HSROC:* Rutter and Gatsonis 2001) for diagnostic test accuracy meta-analyses. The *bivariate* and *HSROC* models are equivalent under most common circumstances, for example when no covariate is included or when the sensitivity (Se) and specificity (Sp) of the bivariate model are adjusted for the same covariates. We used only the *bivariate* model in our analyses due to software implementation technicalities. There are three main reasons to favor a random effects hierarchical bivariate model (Reitsma et al. 2005). First, the main parameters to be estimated - global Se and global Sp - are analyzed jointly because they are negatively correlated (due to the threshold effect). Second, between-study variability is generally high in this type of meta-analysis. Thus, the use of hierarchical random effects models is necessary to appropriately take into account both within and between study variability. Third, the method employs a regression model that naturally allows the inclusion of study-level covariates to perform meta-regressions.

The main assumption of this hierarchical model is at the second level, namely that true values of the logit-transformed Ses and Sps of the individual studies should be independent and follow normal distributions (with unknown means and variances). The main parameters of interest that are estimated are the means of the underlying normal distributions of the Se and Sp values. It is important to use these normal distributions, as a priori the studies have different Se and Sp values due to numerous differences in the study characteristics. Logit-transformed Se and Sp values are difficult to interpret directly. We therefore used the inverse logit transformation of the estimated means to obtain the global Se and Sp values.

Meta-regressions additionally assume homoscedasticity of heterogeneity variances, that is, between-study variances of the logit-sensitivity and logit-specificity parameters must remain approximately constant through the covariate levels. Here, the number of studies was too low to verify these assumptions. However, subgroup analyses were used to confirm meta-regression results without having to assume homoscedasticity. No zero-cell adjustment was needed since the model used binomial distributions to correctly model sampling uncertainty in both Se and Sp.

As mentioned above, a category of cognition was meta-analyzed when it contained at least three studies. These meta-analyses all consisted of a small number of studies (from 3 to 6) with occasionally small sample sizes. For such meta-analyses, the maximum likelihood estimation of the *bivariate* and *HSROC* model parameters often suffer from convergence problems (Paul et al. 2010). Therefore, we chose the *bivariate* Bayesian implementation of the R package, meta4diag (Guo and Riebler 2016), which is based on the Integrated Nested Laplace Approximations method (Rue et al. 2009).

The estimations of the variances and correlation of the joint distribution of Se and Sp values were not precise, because of the small number of studies, and were thus not informative. Since the model uses Bayesian inference, the reported credible intervals (CrI) for global sensitivities and specificities are credible intervals that correspond to the posterior distributions of the parameters. By definition, the estimated parameters lie within the CrIs with a posterior probability of 95% (Robert 2001). In other words, the CrIs must be interpreted to have a 95% certainty of containing the true parameter.

### Exploration of Sources of Heterogeneity

We considered mean age and the length of follow-up of study participants to be potential sources of heterogeneity, as these variables could influence the pooled estimates. These analyses were performed through meta-regressions in which these variables were considered to be continuous covariates, one at a time. We also carried out subgroup analyses whenever the CrI of the effect of the covariate on Se or Sp was found to exclude the zero value. These analyses were performed by dividing the studies into two more or less equally numbered subgroups based on the covariate values.

### Sensitivity to Prior Distributions

Vague prior distributions used in Bayesian analyses are generally expected to yield findings close to those obtained with other frequently used methods (Spiegelhalter et al. 1999). However, given the small number of studies and the sparse data involved in our meta-analyses, it was important to verify the sensitivity to prior distributions. The prior distributions that are susceptible to have an influence on the results are those of the heterogeneity variances and the correlation. However, as noted by Paul et al. (2010), the influence of the correlation prior is expected to be less for a data set with a lower correlation. Since we did not observe strong correlations in our data, we left the correlation prior as the default of the meta4diag function: normal distribution with a mean of 0 and a variance of 5. Hence, our sensitivity analyses were performed by varying only the prior distribution of the heterogeneity variances. We took two of the priors suggested by (Paul et al. 2010), namely 1) mildly informative inverse gamma distribution with a shape of 0.25 and a rate of 0.025 and 2) the less informative inverse gamma distribution with both the shape and rate equal to 0.001. The priors for the mean parameters and beta-coefficients of the covariates were also used as the default for both sensitivity and specificity, that is, normal prior with a mean of zero and a large variance (exact value of variance not specified by the authors of the package). The two priors for the heterogeneity variances usually yielded very close results, in which case only one was reported.

### Publication Bias

The number of studies was insufficient to assess publication bias. Fortunately, this type of bias is less expected to occur for reviews of diagnostic test accuracy than for other types of reviews. In fact, Leeflang (2014) suggested that there is little evidence of publication bias for diagnostic test accuracy studies and hence publication bias is less of a concern for diagnostic test accuracy studies than for other types of reviews (Leeflang 2014).

### Subsidiary Analyses Excluding High Risk of Bias Studies

Se and Sp values may be inflated by the inclusion of studies with a high risk of bias (Whiting et al. 2011). We performed subsidiary analyses after removal of all such studies when possible and then compared the results to those obtained when including all studies.

## Results

### Study Selection

**Fig. 1:**
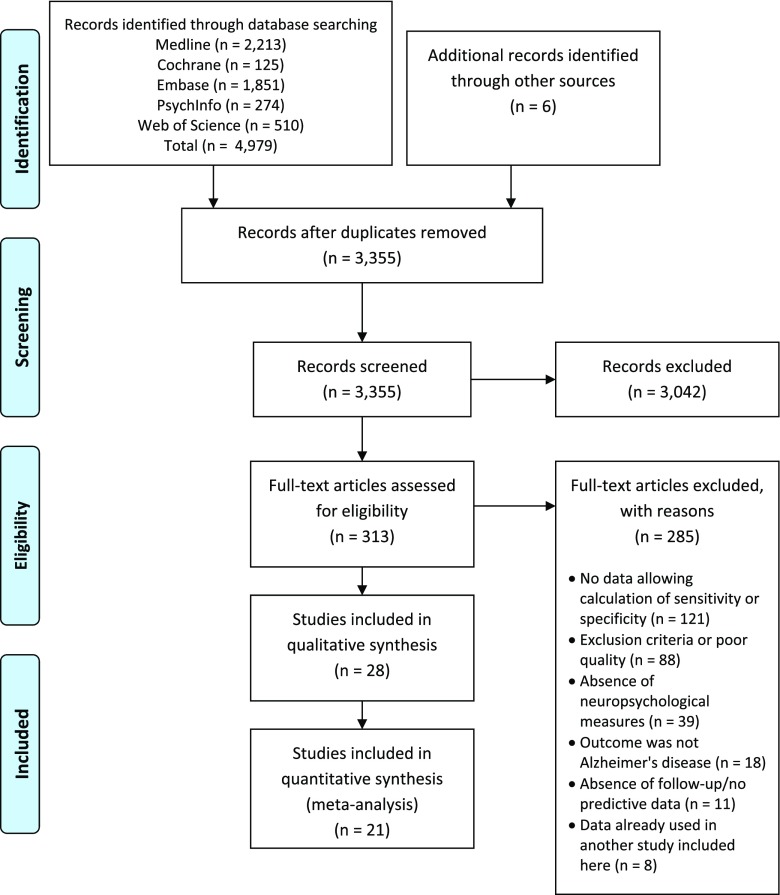
PRISMA 2009 Flow Diagram

The search strategy identified 4979 citations from electronic databases and six citations from hand searching. After adjusting for duplicates, 3355 citations remained in the pool of papers. After screening titles and abstracts, 313 citations were potentially eligible and full reports were retrieved and analyzed. We then excluded 285 studies which did not meet criteria based on a detailed examination of the full text, thus leaving 28 studies for the systematic review and meta-analysis. All were included in the systematic review. Of these, 21 met criteria described above to be included in the meta-analysis (Fig. 1).

### Study Characteristics

The included studies and their characteristics are shown in Table 1. Among the 28 selected studies (references listed in Table 1), 22 were prospective, four were retrospective, and two used data from the Alzheimer’s Disease Neuroimaging Initiative. The studies on which the review is based involved a total of 2365 participants with MCI. Participants were followed over a period of 12 to 60 months (mean follow-up: 31 +/−14 months). Among the enrolled MCI participants, 916 (38.7%) later fulfilled criteria for a diagnosis of AD. The mean conversion rate varied from 6 to 39% per year based on studies that used a prospective study design.

**Table 1:** Characteristics of included Studies

Study	N	Type of MCI/criteria	Outcome	Follow-up in months	N progressors (%)	Age of progressors M (SD)	% female for progressors	Education of progressors	Neuropsychological tests included in the analysis	Type of study
(Ahmed et al. 2008)	18	aMCI	AD	12	7 (39)	71.7 (6.8)	n.r.	11.9 (1.6)	● Addenbrooke’s Cognitive Examination ● Famous buildings, Associative Learning Battery ● Patterns, Associative Learning Battery ● CANTAB paired associate learning (short version) ● Graded naming test ● Animal fluency ● Trail making test, Part B ● Addenbrooke’s Cognitive Examination + CANTAB paired associate learning	LP
(Albert et al. 2001)	114	QAD	AD	36	23 (19)	73	48	n.r.	● Trail making test, Part B + Figures WMS, Immediate recall + Self Ordering Test, total score	LP
(Anchisi et al. 2005)	48	aMCI	AD	12 (median)	14 (29)	71.1 (3.9)	64	9.1 (5.0)	● CVLT, long delay recall	LP
(Arnaiz et al. 2001)	20	MCI, GDS = 3	AD	33.6 (14.6)	9 (45)	64.9 (8.3)	33	11.9 (2.2)	● Block design, WAIS-R	LP
(Babins et al. 2008)	8^a^	aMCI	AD	58.8 (13.6)	41 *(50)*	77.6 (5.7)	n.r.	11.54 (3.6)	● Clock drawing test, 18-point scoring system	LP
(Belleville et al. 2014b)	92	aMCI	cognitive decline and AD	30.81 (20)	49 AD +10 decliners MCI (55)	70.5 (7.9)	60	14.5 (4.4)	● Macro Text, delayed recall ● RL/RI 16, free delayed recall ● Alpha span, items alpha recalled ● BORB, line judgement ● BORB, object decision ● D.O.80 ● Combination Macro Text, delayed recall + Free recall of words + D.O.80 + BORB line judgement + BORB object decision +alpha-span	LP
(Buchhave et al. 2008)	147	aMCI	AD, VD and other dementia	62.4	63 (43)	74.6 (6.1)	68	n.r.	Cube copying, total score	LP
(Defrancesco et al. 2013)	60 ^a^	aMCI	AD	18.3 (7.1)	31 *(52)*	76.3 (6.7)	77	10.4 (3.5)	Combination CERAD word list recall + MMSE orientation	LR
(Didic et al. 2013)	26	sd-aMCI	AD	42 (22.2)	15 (58)	71.8 (6.0)	40	11	● FCSRT, free recall ● FCSRT, total recall ● FCSRT, total delayed recall ● Logical Memory WMS-III, Delayed recall ● Logical Memory WMS-III, Recognition ● Knowledge Public Events, free recall ● Knowledge Public Events, total recall ● Rey’s Figure, delayed recall ● DMS48, immediate recognition ● DMS48, delayed recognition ● Face recognition WMS-III ● Combination Logical Memory WMS-III, delayed recall + DMS48, immediate recognition	LP
(Dierckx et al. 2009)	31	sd-aMCI	AD	17 (2)	7 (23)	76.7 (4.9)	29	12.7 (3.3)	● MISplus, total delayed recall ● Visual association test, total	LP
(Eckerstrom et al. 2013)	42^a^	MCI (GDS = 3)	dementia and AD	24	13 (31%)	70.0 (6.5)	69	10.0 (2.9)	● RAVLT delayed recall ● Boston naming test ● VOSP Silhouettes	LR
(Ewers et al. 2012)	130	aMCI	AD	39.6^**b**^	58	74.6 (7.3)	33	n.r.	● RAVLT, delayed recall ● RAVLT, immediate recall^@^ ● RAVLT, delayed recognition ● Digit span, total score ● Trail making test, Part B ● Trail making test, Part B^b^ ● Category (vegetables) fluency	ADNI
(Flicker et al. 1991)	32	MCI (GDS = 3)	GDS decline and AD	25,32+/−1,08	16 (50) + 7 decliners	n.r.	n.r.	n.r.	● Object function recognition ● Object identification	LP
(Gallagher et al. 2010)	182^c^	MCI	AD	26 (17.5)	75 (41)	73.9 (5.9)	53	n.r.	● Combination DWR, free recall + categoryfluency ● DWR-extended, free recall^c^ ● DWR-extended, recognition ^c^ ● CAMCOG, total score ^c^ ● CAMCOG, orientation ^c^ ● CAMCOG, perception ^c^ ● CAMCOG, category fluency ^c^ ● CAMCOG, letter fluency ^c^ ● Boston Naming test ^c^	LR
(Gallagher et al. 2010)									● Combination DWR free recall + DWR recognition	
(Galton et al. 2005)	29	QD	AD	24 (5.4)	11 (35)	70.9 (8.9)	46	n.r.	● Logical Memory WMS-R, immediate recall ● Logical Memory WMS-R, delayed recall ● Warrington’s recognition memory tests, short recognition memory test for words ● Warrington’s recognition memory tests, short recognition memory test for faces ● Doors test, total score ● Addenbrooke’s Cognitive Examination ● ADAS-Cog total ● Category fluency ● Graded naming test ● Cambridge Semantic Battery, Picture naming (64)	LP
(Griffith et al. 2006)	49^d^	sd-aMCI	AD	24	13 (26)	70.2 (6.7)	77	13.5 (1.9)	● Visual Reproduction WMS-III, percent retention ● Combination Dementia Rating Scale, initiation/perserveration + Visual Reproduction, percent retention	LP
(Irish et al. 2011)	15	aMCI	AD	22.4 (9.5)	6 (38)	71.8^e^ (6.8)	38^e^	13.8^e^ (4.7)	● Face name association task, free delayed recall of names	LP
(Kluger et al. 1999)	71	MCI ^f^ (GDS = 3)	Decline^g^	45.6^f^ (26.4)	47 (66)	73.0^f^ (9.1)	61^f^	13.4^f^ (3.3)	● Guild Paragraph, delayed recall	LR
(Lekeu et al. 2010)	34	aMCI	AD	26.8	17 (50)	72.0 (5.9)	71	10.8 (2.5)	● Rey’s figure, delayed recall	LP
(Marcos et al. 2006)	82	aMCI	AD	36	38 (46)	77.6 (6.1)	68	84% <10 years	● Blessed Dementia scale ● MMSE ● CAMCOG, total ● CAMCOG, perception ● CAMCOG, orientation ● Combination CAMCOG global score + CAMCOG memory + CAMCOG perception	LP
(Mitchell et al. 2009)	82	MCI	AD	24	35 (41)	n.r.	n.r.	n.r.	● Combination Addenbrooke’s Cognitive Examination and/or CANTAB paired associate learning	LP
(Perri et al. 2007a)	190	sd-aMCI	AD	24	79 (42)	73,2+/−5,5	56	7,5+/−3,2	● Unrelated word list, immediate recall ● Related word list, immediate recall ● Unrelated word list, delayed recall ● Related word list, delayed recall ● Prose recall, immediate recall ● Prose recall, delayed recall ● Rey’s figure, immediate recall ● Rey’s figure, delayed recall ● CDR, mean sum boxes	LP
(Richard et al. 2013)	181	aMCI	AD	38.9	81 (45)	74.4 (7.4)	38	15.6 (3.0)	● RAVLT Total recall^a^	ADNI
(Sarazin et al. 2007)	217	aMCI	AD	31 (10.5)	59 (26)	74.8 (4.1)	54	n.r.	● FCSRT, delayed free recall ● FCSRT, total delayed recall ● FCSRT, free recall ● FCSRT, total recall ● Similarities WAIS-R ● Benton visual retention test ● Double task Baddeley ● Serial digits ordering tests ● Digit symbol test WAIS-R ● Stroop test ● Trail Making test-Part A ● Trail Making test-Part B ● Naming-Deno 100 ● Letter (S) fluency ● Category (fruit) fluency	LP
(Tabert et al. 2006)	115^h^	MCI	AD	21 (15)	35 (30)	72.7 (7.2)	60	13.9 (4.5)	● Combination SRT, total immediate recall + Digit symbol WAIS-R	LP
(Tierney et al. 1996)	123	CI	AD	24	29 (24)	73.9 (6.7)	n.r.	13.5 (3.0)	● RAVLT, delayed recall + Mental control WMS^c^	LP
(Venneri et al. 2011)	25	MCI	AD	36	11 (44)	72.45 (5.07)	36%	8.64 (4.58)	● Category fluency ● Raven’s progressive matrices ● Paired associate Learning	LP
(Visser et al. 2001)	67	MCI	AD	60	23 (67)	70.1 (6.6)	52	10.8 (3.4)	● RAVLT, delayed recall	LP

*N*, sample size of MCI included in the meta-analysis; *MCI*, Mild cognitive impairment; *M*, Mean; *SD*, Standard deviation; *aMCI*, Amnestic MCI including single and multi-domain types; *AD*, Alzheimer’s disease; *n.r.*, Not reported; *LP*, Longitudinal prospective study; *QAD*, Questionable AD; *WMS*, Wechsler memory scale (III = third version, R = revised); *CVLT*, California verbal learning test; *GDS*, Global deterioration scale; *WAIS-R*, Wechsler adult intelligence scale-revised; *RL/RI 16*, French version of the free and cued recall task; *BORB*, Birmingham object recognition battery; *MEMO-Text*, Text memory; *D.0.80*, Denomination 80 items; *VD*, Vascular dementia; *LR*, Longitudinal retrospective study; *CERAD*, Consortium to establish a registry for Alzheimer’s Disease; *MMSE*, Mini-mental state examination; *sd-aMCI*, Single domain amnestic MCI; *CDR*, Clinical Dementia rating scale; *FCSRT*, Free and Cued selective reminding test, *DMS48*, Delayed matching to sample − 48 items; *MISplus*, Memory impairment screen plus; *RAVLT*, Rey auditory verbal learning test; *VOSP*, Visual object and space perception; *ADNI*, Alzheimer’s disease neuroimaging initiative; *DWR*, Delayed word recall; *CAMCOG*, Cambridge cognitive examination; *QD*, Questionable dementia; *ADAS-cog*, Alzheimer’s disease assessment scale cognitive section, *SRT*, Buscke selective reminding test; *CI*, Cognitively impaired

^@^Data not included because used in another study

^a^Data from convenience sample

^b^Data analyzed for follow-up = 24 or 36 months

^c^Sub-analysis was made for a subgroup of 106 participants with complete 3 year follow-up, N converters = 64 (60%)

^d^Among the 49 participants, 11 converted at the 1 year follow-up (mean time = 13.04 +/− 2.1)

^e^Demographic data from all MCI participants

^f^Demographic data from participants with GDS = 1–3 at baseline

^g^GDS = 4 + AD criteria

^h^Sub-sample from the total MCI population (*n* = 148) with mean time to conversion 21+/−15 months, n converters = 39

Twenty-one of the 28 studies used Petersen’s criteria to identify MCI at entry, consisting of subjective cognitive complaint, evidence for cognitive impairment (relative to age and education), preservation of general functional abilities, and the absence of diagnosed dementia. Among these 21 studies, 12 included both single and multiple domain amnestic MCI (aMCI), four included only single-domain amnestic MCI (sd-MCI), and five included amnestic and non-amnestic MCI. Four studies used the Global Deterioration Scale (GDS) to identify MCI (i.e., those with a score of 3 on the GDS). Three studies used other terminologies to describe their patients. Albert et al. (2001) selected participants with questionable Alzheimer’s disease (QAD), defined as those with a score of 0.5 for the CDR (Albert et al. 2001). Galton et al. selected participants with “questionable dementia,” defined by the presence of subjective complaints of memory impairment substantiated by an informant, (b) normal activities of daily living, and (c) absence of dementia as evident from an MMSE score of ≥23, and a score of 0.5 on the CDR (Galton et al. 2005). Finally, Tierney et al., selected patients with a three-month history of symptomatic memory problems that interfered with daily functioning (GDS 2 or 3), no diagnosis of dementia, and an MMSE ≥24 or a score ≥ 123 on the Mattis Dementia Rating Scale (Mattis 1988; Tierney et al. 1996).

The mean age at entry for those who progressed to AD ranged from 64.9 to 77.6 years (mean age = 71.8 years) versus 59.3 to 75 years (mean age = 69.8 years) for non-progressors. The percentage of females in the group of progressors varied from 33 to 77% (overall percentage = 54%) vs 32 to 72% in the group of non-progressor MCI (overall percentage = 51%). Mean years of education of participants who progressed to AD varied from 7.5 to 15.6 (mean = 12.1 years) vs 7.7 to 15.9 (mean = 12.6 years) for non-progressors. All included studies used the NINCDS-ARDRA criteria as their outcome for AD.

The studies reported predictive values for a total of 61 neuropsychological tests. Sixteen articles reported the predictive values of individual neuropsychological tests, five reported the predictive value of a combination of several neuropsychological tests, and seven included both individual tests and combinations of tests.

### Risk of Bias within Studies

Table 2 shows the risk of bias associated with the included studies. Ten studies showed a low risk of bias, eight a moderate risk of bias, and ten a high risk of bias (all references listed in Table 2). The most frequent limitation comes from the selection process of the sample, which may not be representative of the population of interest. For example, Eckerstrom et al. selected, a posteriori*,* a sub-sample of non-progressors to match the sample of progressors. Other studies only selected participants who previously underwent particular examinations (e.g., PET scan in Arnaiz et al. 2001 or a CSF examination in Richard et al. 2013). Griffith et al., included participants who were taking cholinesterase inhibitors (13 of 49 MCI). Other studies did not include sufficient details about the selection process (Babins et al. 2008). Many studies relied on a small sample size (< 40 participants: Ahmed et al. 2008; Arnaiz et al. 2001; Didic et al. 2013; Dierckx et al. 2009; Flicker et al. 1991; Galton et al. 2005; Irish et al. 2011; Lekeu et al. 2010; Venneri et al. 2011) or showed a higher progression rate than expected (Gallagher et al. 2010; Irish et al. 2011; Lekeu et al. 2010).

**Table 2:** Risk of bias within studies

Study	Patient selection *(variation in participant selection, conversion rate, MCI definition)*	Study design appropriate *(retrospective or prospective, independence between diagnostic and predictive assessment)*	Flow and follow-up *(Follow-up sufficiently long, Loss to follow-up explained)*	Reference standard *(outcome clearly defined and suitable diagnostic procedure)*	Prognostic variables *(predictive tests well described)*	Analyses *(Identification of an optimal 5subset and threshold specified independently from dataset)*	Risk of Bias
(Ahmed et al. 2008)	Yes, but small sample	Yes	Yes but short follow-up	Yes	Yes	No regression, use independent threshold	L
(Albert et al. 2001)	No, lots of exclusion criteria (15% eligibility), QAD based only on CDR (seem less impaired than MCI)	Yes	Yes	Yes	Yes	Yes stepwise discriminant function, no specified threshold	M
(Anchisi et al. 2005)	Yes	No independence between diagnostic and predictive assessments	Yes but short follow-up	Yes	Yes	ROC curve analysis, threshold calculated on sample	H
(Arnaiz et al. 2001)	No, small sample, no details about exclusion criteria (seems strict, e.g., having PET examination).	No, absence of independence between diagnostic and predictive batteries	Yes	Yes	Yes	Yes, logistic regression, no specified threshold	M
(Babins et al. 2008)	No, not sure of selection process (50% progressors, maybe a posteriori selection of subsample)	Not sure if retrospective or not.	Yes	Yes	Yes	Yes, ROC curves analysis, but threshold calculated on sample	H
(Belleville et al. 2014b)	Yes	Yes	Yes	Yes, but pooled together MCI decliners and converters	Yes	Yes, backward logistic regression but threshold calculated on sample	L
(Buchhave et al. 2008)	Yes	Yes (MMSE used in both assessments was not included in systematic review)	Yes	Yes	Yes	ROC curves, hreshold calculated on sample	L
(Defrancesco et al. 2013)	No, convenience sample (selection of participants to maximize number of progressors)	No, retrospective study, not sure of independence between diagnostic and predictive assessments	Yes	Yes	Yes	Yes, stepwise logisitc regression, no specified threshold	H
(Didic et al. 2013)	Not sure, only sd-a MCI, small sample	Yes	Yes	Yes	Yes	ROC curves, threshold calculated on sample	L
(Dierckx et al. 2009)	No, sd-aMCI, no information about exclusion criteria, small sample	Yes	Yes	Yes	Yes	Yes, binary logistic regression analysis, but ROC curves, threshold calculated on sample	H
(Eckerstrom et al. 2013)	No, selection of participants who had MRI with a specific scanner, (non- progressors were added to match the converted sample)	No, retrospective	Yes	Yes	Yes	Yes, Partial Least Squares Discriminant Analysis but ROC curves, no specified threshold	H
(Ewers et al. 2012)	Yes	Yes (selection from ADNI database, identical to whole sample)	Yes	Yes	Yes	Yes, logistic regression, no specified threshold	L
(Flicker et al. 1991)	Yes, but relatively small sample	Not sure of independence between diagnostic and predictive assessments	Yes	No, but pool together GDS decline and AD (not specific AD) complete diagnostic evaluations at FU only on GDS decliners	No	Lack of details, no specified threshold	H
(Gallagher et al. 2010)	Yes, but high conversion rate	No, retrospective. Not sure of independence between diagnostic and predictive assessments	Yes	Yes	Yes	Yes, cox proportional hazards regression, ROC curves threshold calculated on sample	H
(Galton et al. 2005)	Yes, but no memory deficit needed in cognitive deficit criteria, small sample	Yes	Yes	Yes	Yes	Separate discriminant function for each variable, no specified threshold	L
(Griffith et al. 2006)	No, lots of exclusion, 13/49 participants were taking medication for memory problems	Yes, but not sure of independence between diagnostic and predictive assessments	Yes	Yes	Yes	Yes, stepwise discriminant function analysis, threshold calculated on sample	H
(Irish et al. 2011)	Yes, but very small sample, high conversion rate	Yes	Yes	Yes	Yes	Yes, regressions models but threshold calculated on sample	M
(Kluger et al. 1999)	No, very restrictive exclusion criteria and definition with GDS	No, retrospective study	Yes	Yes	Yes	Yes, regressions analyses but threshold calculated on sample	H
(Lekeu et al. 2010)	Yes but, high conversion rate, small sample	Yes, but not sure of independence between diagnostic and predictive assessments	Yes	Yes	Yes	Yes, regression analyses but ROC curves threshold calculated on sample	M
(Marcos et al. 2006)	Yes	No, absence of independence between diagnostic and predictive assessments	Yes, but no explanation about loss to follow-up	Yes	Yes	Not sure, pool together global camcog and camcog subtest while they are correlated, ROC curves threshold calculated on sample	M
(Mitchell et al. 2009)	Yes	Yes	Yes	Not sure (MMSE and CDR used to diagnose conversion)	Yes	Yes, stepwise discriminant analysis, threshold based on control group data	M
(Perri et al. 2007a)	Yes, but sd-aMCI	Yes	Yes	Yes	Yes	Yes, logistic regression, threshold based on normative data	L
(Richard et al. 2013)	Not sure, selection of participants who had imaging and CSF data	Yes (data from ADNI database)	Yes	Yes	Yes	Yes, stepwise forward analysis, but ROC curves, threshold calculated on sample	L
(Sarazin et al. 2007)	Yes	Yes	Yes	Yes	Yes	Yes, logistic regression, but but ROC curves, threshold calculated on sample	L
(Tabert et al. 2006)	No, pool together people with and without objective cognitive deficits	No, absence of independence between diagnostic and predictive assessments	Yes	Yes	Yes	Yes, Cox regressions, logistic regressions, no specified thresholds	H
(Tierney et al. 1996)	Not sure, lots of exclusion criteria, use GDS and DRS as criteria for inclusion	Yes	Yes	Yes	Yes	Yes, logistic regression, no specified threshold	L
(Venneri et al. 2011)	Yes but small sample	Yes, but absence of independence between diagnostic and predictive assessments	Yes	Yes	Yes	Threshold based on control group data	M
(Visser et al. 2001)	Yes	No, restrospective, not sure of independence between diagnostic and predictive batteries	Yes	Yes	Yes	Yes, logistic regression analysis, no specified threshold	M

*MCI*, Mild cognitive impairment; *follow-up*, follow-up; *L*, Low risk of biais; *M*, Moderate risk of bias; *H* , High risk of bias; *QAD*, Questionable Alzheimer’s disease; *CDR*, Clinical dementia rating scale; *ROC curve*, Receiver operating characteristic curve; *PET*, Positron emmision tomography; *MMSE*, Mini-mental State examination; *MRI*, Magnetic resonnance imagery; *GDS*, Global deterioration scale; *sd-aMCI*, Single domain amnestic MCI; *DRS*, Dementia rating scale; *CSF*, Cerebrospinal fluid; *ADNI*, Alzheimer’s disease neuroimaging initiative

Other aspects of the study design raised issues related to quality, albeit less frequently. For example, five studies did not ensure strict independence between the diagnostic battery and the predictive battery (Anchisi et al. 2005; Arnaiz et al. 2001; Marcos et al. 2006; Tabert et al. 2006; Venneri et al. 2011). Thus, either some of the neuropsychological tests included in the predictive battery were also used to make the baseline or follow-up diagnosis of the participants or the staff administering the diagnostic battery were not blind to the participants’ performance on the predictive battery. Six studies did not provide sufficient information to determine whether the diagnostic and predictive assessments were independent with certainty (Defrancesco et al. 2013; Flicker et al. 1991; Gallagher et al. 2010; Griffith et al. 2006; Lekeu et al. 2010; Visser et al. 2001).

Most studies had a duration of follow-up longer than 12 months and most reported the reasons for loss to follow-up. Only two studies had a relatively short follow-up (12 months), which likely increased the risk of false negatives (Ahmed et al. 2008; Anchisi et al. 2005). Most studies used appropriate procedures for diagnosis. Two studies pooled patients who converted to AD and patients who showed significant cognitive decline (Belleville et al. 2014b; Flicker et al. 1991) and thus compared “stable MCI” to “decliners”. Flicker et al. administrated the reference standard battery for AD diagnosis only to patients who showed decline on the GDS. Thus, this study presents a “partial bias verification”. As per our inclusion criteria, all the predictive tests were well described or relied on tests that are well known and for which an extensive description is available from the literature.

Most studies used ROC curves to calculate optimal cut-offs for the tests and determine predictive accuracy. Twenty studies used regression analyses to assess and compare the predictive accuracy of different tests.

### Results from the Systematic Review

A summary of the predictive accuracy values for single domains is presented in Table S3 (*Supplementary material*). The sensitivities observed from individual studies ranged from 0.18 to 1 and the specificities ranged from 0 to 1. A very large number of tests show very good (≥ 0.70) sensitivity, very good specificity, or both. The tests which obtained the sensitivity and specificity values in the excellent range (≥ 0.90) are shown in Table 3. The table indicates predictive values (number of true positives, true negatives, false positives, false negatives, sensitivity, specificity, and accuracy) for each cognitive domain category. The cut-off values of the tests used in the studies are included, if available. Individual tests showed excellent specificity more often than excellent sensitivity. The tests with best overall accuracy (≥ 0.90) are shown in Table 4. Five individual tests and one global measure had excellent overall predictive value, implying that they combine very high specificity and sensitivity. The tests were 1) the Guild Paragraph, delayed recall, 2) the RAVLT, delayed Recall, 3) Face-name association task, free delayed recall of names, 4) Object function recognition, 5) VOSP Silhouettes, and the ACE Addenbrooke’s Cognitive Examination.

**Table 3:** Individual tests with excellent specificity or sensitivity, grouped by cognitive domain

	TP	FP	FN	TN	SE	SP	ACC	Cut-off
Verbal episodic memory								
Verbal immediate recall**								
Logical memory, immediate recall (Galton et al. 2005)*	10	4	1	14	0.91	0.78	0.83	n.r.
FCSRT, free recall (Sarazin et al. 2007)*^a^	42	13	17	145	0.71	0.92	0.86	17
World-list cued immediate recall with oriented encoding								
FCSRT, total recall (Didic et al. 2013)	10	1	5	10	0.67	0.91	0.77	37
FCSRT, total recall (Sarazin et al. 2007)^a^	47	16	12	142	0.80	0.90	0.87	40
paragraph delayed recall**								
Logical memory, delayed recall (Galton et al. 2005)*	10	4	1	14	0.91	0.78	0.83	n.r.
Logical memory, delayed recall (Didic et al. 2013)*	11	0	4	11	0.73	1.00	0.85	6
Guild Paragraph, delayed recall (Kluger et al. 1999)*	45	4	2	20	0.96	0.83	0.92	n.r.
Word-list free delayed recall with non oriented encoding**								
RAVLT, delayed recall (Eckerstrom et al. 2013)* ^c^	12	1	1	20	0.92	0.94	0.94	n.r.
RAVLT, delayed recall (Visser et al. 2001)*	17	3	6	41	0.74	0.93	0.87	n.r.
Word-list free delayed recall with oriented encoding**								
CVLT, long delay free recall (Anchisi et al. 2005)*	13	14	1	20	0.93	0.59	0.69	7
FCSRT, delayed free recall (Sarazin et al. 2007)* ^a^	45	15	14	143	0.76	0.91	0.87	6
Face-name association task, free delayed recall of names (Irish et al. 2011)*	6	1	0	8	1.00	0.86	0.92	3
Word-list cued delayed recall with oriented encoding **								
FCSRT, total delayed recall (Didic et al. 2013)*	9	1	6	10	0.60	0.91	0.73	12
MISplus total delayed recall free&cued (Dierckx et al. 2009)*	5	2	2	22	0.71	0.92	0.87	2
Paragraph recognition								
Logical memory WMS-III recognition (Didic et al. 2013)	5	0	6	9	0.45	1.00	0.70	18
Visual episodic memory								
Immediate recall								
Rey’s figure, immediate recall (Perri et al. 2007b)	14	9	65	102	0.18	0.92	0.61	n.r.
Delayed recall**								
Visual Reproduction, percent retention (Griffith et al. 2006)*	10	3	3	33	0.77	0.91	0.88	26
Visual recognition								
DSM48, immediate recognition (Didic et al. 2013)	12	1	3	10	0.80	0.91	0.85	89
Face recognition WMS-III, immediate scaled score (Didic et al. 2013)	14	6	1	5	0.93	0.45	0.73	12
Associative memory								
Paired Associate Learning (Venneri et al. 2011)	11	14	0	0	1.00	0.00	0.44	n.r.
CANTAB paired associate learning short version (Ahmed et al. 2008)	7	5	0	6	1.00	0.55	0.72	14
Visual Association test total (Dierckx et al. 2009)	3	1	4	23	0.43	0.96	0.84	1/6
Patterns, Associative Learning Battery, errors (Ahmed et al. 2008)	7	6	0	5	1.00	0.46	0.67	18
Language								
Naming tests**								
Boston naming test; 60 items (Eckerstrom et al. 2013)* ^c^	13	5	0	16	0.99	0.74	0.85	n.r.
Graded naming test (Ahmed et al. 2008)*	3	1	4	10	0.40	0.91	0.72	14
Tests of semantic knowledge**								
Object function recognition (Flicker et al. 1991)*	20	0	3	9	0.86	1.00	0.90	n.r.
Object identification (Flicker et al. 1991)	13	0	10	9	0.57	1.00	0.69	n.r.
Semantic fluency**								
Category fluency; animals, vegetables & fruits (Gallagher et al. 2010)*	60	22	4	20	0.94	0.48	0.75	36
Category fluency; animals (Ahmed et al. 2008)*	2	0	5	11	0.29	1.00	0.72	11
Category fluency (Venneri et al. 2011)*	10	7	1	7	0.91	0.50	0.68	n.r.
Visuo-constructive functions								
Visuo-spatial tests**								
VOSP Silhouettes (Eckerstrom et al. 2013)*^c^	12	2	1	19	0.92	0.92	0.91	n.r.
Brief and Global measures								
Global measures**								
ACE Addenbrooke’s Cognitive Examination (Ahmed et al. 2008)*	7	5	0	6	1.00	0.55	0.72	88
ACE Addenbrooke’s Cognitive Examination (Galton et al. 2005)*	8	0	3	18	0.73	1.00	0.90	n.r.
CAMCOG, total score (Marcos et al. 2006)*	35	14	3	30	0.92	0.68	0.79	79.5

*TP*, Number of true positive; *FP*, Number of false positive; *FN*, Number of false negative; *TN*, Number of true negative; *SE*, Sensitivity; *SP*, Specificity; *ACC*, Overall accuracy; *n.r.*, Not reported

**Cognitive domains included in a meta-analysis

*data included in a meta-analysis

^a^Age corrected data

^b^Age, gender and education corrected data

^c^Age and education corrected data

**Table 4:** Individual tests with excellent overall accuracy

	TP	FP	FN	TN	SE	SP	ACC	Cut-off
Verbal episodic memory								
Paragraph delayed recall**								
Guild Paragraph, delayed recall (Kluger et al. 1999)*	45	4	2	20	0.96	0.83	0.92	n.r.
Word-list free delayed recall with non oriented encoding**								
RAVLT, delayed Recall (Eckerstrom et al. 2013)* ^c^	12	1	1	20	0.92	0.94	0.94	n.r.
Word-list free delayed recall with oriented encoding**								
Face-name association task, free delayed recall of names (Irish et al. 2011)*	6	1	0	8	1	0.86	0.92	3
Language								
Tests of semantic knowledge**								
Object function recognition (Flicker et al. 1991)*	20	0	3	9	0.86	1.00	0.90	n.r.
Visuo-constructive functions								
Visuo-spatial tests**								
VOSP Silhouettes (Eckerstrom et al. 2013)*^c^	12	2	1	19	0.92	0.92	0.91	n.r.
Brief and Global measures								
Global measures**								
ACE Addenbrooke’s Cognitive Examination (Galton et al. 2005)*	8	0	3	18	0.73	1.00	0.90	n.r.

*TP*, Number of true positive; *FP*, Number of false positive; *FN*, Number of false negative; *TN*, Number of true negative; *SE*, Sensitivity; *SP*, Specificity; *ACC*, Overall accuracy; *n.r.*, Not reported

*Data included in a meta-analysis

**Cognitive domains included in a meta-analysis

^a^Age corrected data

^b^Age, gender and education corrected data

^c^Age and education corrected data

Results obtained from combining different neuropsychological tests are provided in Table 4 (*Supplementary material*). Most studies combined episodic memory tests with global measures or non-memory measures. Studies that examined a combination of memory and non-memory tests obtained very high to excellent predictive accuracy, with a few exceptions. Combining episodic memory tests with executive function tests gave very good sensitivities (from 0.74 to 0.77) and excellent specificities (0.83 to 0.94: Albert et al. 2001; Griffith et al. 2006; Tabert et al. 2006; Tierney et al. 1996). The combination of episodic memory with category fluency allowed for excellent sensitivity (0.84) and very good specificity (0.79: Gallagher et al. 2010). One study reported high sensitivity and specificity (0.88 and 0.87, respectively) when combining episodic memory, language, visual perception, and executive function tasks (Belleville et al. 2014b).

Two studies combining an associative memory task (CANTAB Paired associate learning) with a global measure (Addenbrooke’s Cognitive Examination) reported different results. Ahmed et al. reported excellent accuracy (1 and 0.82 for sensitivity and specificity, respectively), whereas Mitchell et al. reported high sensitivity (0.94), but low specificity (0.40). This may be due to the difference in the duration of follow-up (12 versus 24 months) or study sample (18 versus 82 patients). One study reported that combining associative memory tests with language (category fluency) or visuo-constructive tasks provided very high sensitivities (0.73 to 0.91), but low specificities (0.5 to 0.57: Venneri et al. 2011). The same study reported low sensitivity and specificity values (0.64 and 0.5, respectively) for a combination of associative memory, language measures, and visuo-constructive tasks (Venneri et al. 2011). However, this study was carried out with a small sample size (*n* = 25).

Combining episodic memory with global measures generally provided very good to excellent predictive accuracy. The combination of an episodic memory test with a brief measure (MMSE orientation) provided very good sensitivity and excellent specificity (0.74 and 0.9, respectively: Defrancesco et al. 2013). Combining episodic memory with global measures and visual perception showed excellent sensitivity (0.92) and very good specificity (0.73: Marcos et al. 2006). One study that combined episodic memory tests with a global measure (CDR mean sum of boxes) obtained low sensitivity (0.66), but high specificity (0.86: Perri et al. 2007b). The combination of different episodic memory modalities gave variable results ranging from low sensitivity (0.44) and high specificity (0.9: Gallagher et al. 2010) to excellent sensitivity and specificity (1: Didic et al. 2013).

Given the large variability observed in the predictive accuracy obtained from individual tests, the meta-analysis presented below, summarizes predictive accuracy values (sensitivity and specificity) by domain subgroups. We then present the results from meta-regression, which examined the interaction of sensitivity and specificity with the duration of follow-up, as a proxy for the time to diagnosis, and age.

### Results of the Meta-Analyses

Convergence was achieved in all meta-analyses, except one (associative memory), and only two meta-regressions (verbal immediate recall and paragraph delayed recall) had one prior distribution for which convergence failed. In addition, the second prior distribution analysis failed for short follow-up sub-group for semantic fluency.

#### Verbal Episodic Memory

Most tests measuring verbal episodic memory reported very good predictive accuracy (all above 0.7) and there was little variability related to the test condition (e.g., delayed or immediate, with or without oriented encoding, with or without cues at recall) or the particular material (e.g., words or paragraph). The only exceptions were for recognition and word-list cued delayed recall with oriented encoding, which had lower sensitivity values than other verbal memory tests. Predictive accuracy for tests of verbal immediate recall was reported in five studies (Galton et al. 2005; Perri et al. 2007b; Didic et al. 2013; Sarazin et al. 2007; Richard et al. 2013). The combined value was 0.745 for sensitivity (95% CrI: 0.511–0.905) and 0.771 for specificity (95% CrI: 0.581–0.897). Predictive accuracy for paragraph delayed recall was reported in five studies (Didic et al. 2013; Galton et al. 2005; Belleville et al. 2014b; Kluger et al. 1999; Perri et al. 2007b). The combined analysis gave a 0.776 sensitivity (95% CrI: 0.541–0.928) and a 0.794 specificity (95% CrI: 0.517–0.961). Word-list free delayed recall with non-oriented encoding was reported in four studies (Eckerstrom et al. 2013; Ewers et al. 2012; Visser et al. 2001; Perri et al. 2007b). The combined analysis gave 0.742 sensitivity (95% CrI: 0.625–0.868) and a 0.814 specificity (95% CrI: 0.517–0.961). Word-list free delayed recall with oriented encoding was also reported in five studies (Anchisi et al. 2005; Gallagher et al. 2010; Sarazin et al. 2007; Belleville et al. 2014b; Irish et al. 2011). The combined analysis gave 0.781 sensitivity (95% CrI: 0.704–0.867) and a 0.797 specificity (95% CrI: 0.651–0.893). Word-list cued delayed recall with oriented encoding was reported in three studies (Sarazin et al. 2007; Didic et al. 2013; Dierckx et al. 2009). The combined analysis gave 0.676 sensitivity (95% CrI: 0.526–0.796) and 0.896 specificity (95% CrI: 0.822–0.947). Word-list recognition was reported in three studies (Galton et al. 2005; Ewers et al. 2012; Gallagher et al. 2010). The combined analysis gave 0.547 sensitivity (95% CrI: 0.428–0.671) and 0.789 specificity (95% CrI: 0.602–0.927).

#### Visual Episodic Memory

Four studies reported predictive accuracy for visual episodic memory tests (Didic et al. 2013; Griffith et al. 2006; Lekeu et al. 2010; Perri et al. 2007b). The combined analysis showed a relatively low sensitivity, 0.676 (95% CrI: 0.413–0.889), but very good specificity, 0.847 (95% CrI: 0.722–0.913).

#### Language

The predictive accuracy provided by language tests was relatively high with values just below 0.7 for sensitivity and somewhat higher for specificity. Predictive accuracy for naming tests was reported in six studies (Eckerstrom et al. 2013; Gallagher et al. 2010; Ahmed et al. 2008; Galton et al. 2005; Belleville et al. 2014b; Sarazin et al. 2007). The analysis gave a combined sensitivity score of 0.699 (95% CrI: 0.51–0.868) and a combined specificity score of 0.707 (95% CrI: 0.621–0.813). Data for tests of semantic knowledge was reported in three studies (Didic et al. 2013; Sarazin et al. 2007; Flicker et al. 1991). The analysis gave a combined sensitivity score of 0.703 (95% CrI: 0.442–0.906) and a higher combined specificity of 0.814 (95% CrI: 0.631–0.984). Tests of semantic fluency were examined in six studies (Gallagher et al. 2010; Galton et al. 2005; Ahmed et al. 2008; Ewers et al. 2012; Sarazin et al. 2007; Venneri et al. 2011). The combined sensitivity score was 0.708 (95% CrI: 0.468–0.875) and the combined specificity score 0.7 (95% CrI: 0.539–0.836).

#### Executive Functions

This domain yielded relatively low predictive accuracy values with better specificity than sensitivity. Three studies reported predictive accuracy for switching tests (Trail test; Ahmed et al. 2008; Sarazin et al. 2007; Ewers et al. 2012). The analysis gave a combined sensitivity score of 0.541 (95% CrI: 0.314–0.701) and a somewhat higher combined specificity score of 0.679 (95% CrI: 0.542–0.797). Three studies reported predictive accuracy for working memory tests (Ewers et al. 2012; Sarazin et al. 2007; Belleville et al. 2014b). The combined sensitivity score was 0.599 (95% CrI: 0.463–0.724) and the specificity was again slightly better with a combined score of 0.679 (95% CrI: 0.573–0.747).

#### Visuo-Constructive Functions

Four studies reported predictive accuracy for visuo-spatial tests (Gallagher et al. 2010; Marcos et al. 2006; Belleville et al. 2014b; Eckerstrom et al. 2013). The combined sensitivity score was 0.68 (95% CrI: 0.366–0 .914) and the combined specificity score was 0.749 (95% CrI: 0.613–0.873). Three studies reported data from visuo-constructive tasks (Babins et al. 2008; Buchhave et al. 2008; Arnaiz et al. 2001). Their combined value was 0.637 (95% CrI: 0.471–0.778) for sensitivity and 0.643 (95% CrI: 0.496–0.775) for specificity.

#### Brief or Global Measures

Four studies reported predictive accuracy for brief or global cognitive measures and their combined predictive accuracy was quite high, both in terms of sensitivity and specificity (Ahmed et al. 2008; Galton et al. 2005; Gallagher et al. 2010; Marcos et al. 2006). The analysis gave a combined sensitivity score of 0.852 (95% CrI: 0.73–0.946) and a combined specificity score of 0.757 (95% CrI: 0.468–0.966).

#### Analyses Excluding the High Risk of Bias Studies

The results of the meta-analyses performed after removal of the 10 studies with a high risk of bias are presented on the right-hand side of Table 5. Three test categories did not require re-analysis as they did not contain any high risk of bias studies (verbal immediate recall, switching tests, and working memory tests). Specificity and sensitivity scores obtained when excluding high-risk studies generally remained close to the values obtained from the initial analyses. In three cases, sensitivities that were above 0.70 fell below that score when excluding high-risk studies: paragraph delayed recall, naming, and semantic fluency. However, for naming and semantic fluency, lower sensitivities were partially compensated by slightly higher specificities. In the case of the visuo-constructive tasks category, the specificity increased from below 0.70 to very good (above 0.70) when excluding high-risk studies. Four test categories could not be re-analyzed because they ended up with only two studies: word-list cued delayed recall with oriented encoding, word-list recognition, semantic knowledge and visuo-spatial tests. No sensitivity analyses were performed for the subgroups as they all contained only three studies.

**Table 5:** Meta-analyses

Cognitive domains	All studies			High risk of bias studies excluded		
	N	Sensitivity [CrI]	Specificity [CrI]	N	Sensitivity [CrI]	Specificity [CrI]
Verbal episodic memory						
Verbal immediate recall	5	0.745^a^ [0.511–0.905]	0.771^a^ [0.581–0.897]	No high risk of bias study		
Paragraph delayed recall	5	0.776 [0.541–0.928]	0.794 [0.675–0.914]	4	0.636 [0.510–0.808]	0.795 [0.623–0.953]
Word-list free delayed recall with non oriented encoding,	4	0.742 [0.625–0.868]	0.814 [0.517–0.961]	3	0.707 [0.584–0.815]	0.744 [0.354–0.950]
Word-list free delayed recall with oriented encoding	5	0.781 [0.704–0.867]	0.797 [0.651–0.893]	3	0.765 [0.647–0.891]	0.869 [0.717–0.944]
Word-list cued delayed recall with oriented encoding	3	0.676 [0.526–0.796]	0.896 [0.822–0.947]	2	Not analyzable	
Word-list recognition	3	0.547 [428–0.671]	0.789 [0.602–0.927]	2	Not analyzable	
Visual episodic memory						
Delayed recall	4	0.676 [0.413–0.889]	0.847 [0.722–0.913]	3	0.650 [0.320–0.912]	0.814 [0.624–0.904]
Language						
Naming tests	6	0.699 [0.51–0.868]	0.707 [0.621–0.813]	4	0.571 [0.458–0.683]	0.742 [0.622–0.887]
Tests of semantic knowledge	3	0.703^b^ [0.442–0.906]	0.814 ^b^ [0.631–0.984]	2	Not analyzable	
Semantic fluency	6	0.708 [0.468–0.875]	0.7 [0.539–0.836]	5	0.598 [0.425–0.779]	0.744 [0.565–0.891]
Executive functions						
Switching tests	3	0.541 [0.314–0.701]	0.679 [0.542–0.797]	No high risk of bias study		
Working memory tests	3	0.599 [0.463–0.724]	0.667 [0.573–0.747]	No high risk of bias study		
Visuo-constructive functions						
Visuo-spatial tests	4	0.68 [0.366–0.914]	0.749 [0.613–0.873]	2	Not analyzable	
Visuo-constructive tasks	4	0.637 [0.471–0.778]	0.643 [0.496–0.775]	3	0.561 [0.399–0.722]	0.705 [0.557–0.823]
Brief/global measures	4	0.852^c^ [0.73–0.946]	0.757 ^c^ [0.468–0.966]	3	0.899 [0.721–0.981]	0.851 [0.294–1.00]

Global estimate (median of posterior distribution) and 95% credible intervals (CrI) of sensitivity and specificity of tests assessing cognitive domains. N indicates the number of study results included in each category

^a^For these data, an alternative prior distribution gave the following results: Sensitivity = 0.747 CrI [0.49–0.915], Specificity = 0.772 [0.564–0.904]

^b^For these data, an alternative prior distribution gave the following results: Sensitivity = 0.708 CrI [0.385–0.927], Specificity = 0.834 [0.478–1]

^c^For these data, an alternative prior distribution gave the following results: Sensitivity = 0.853 CrI [0.705–0.923], Specificity = 0.769 [0.38–0.987]

**Table 6:** Meta-regressions assessing the effect of follow-up duration or age of participants at baseline on sensitivity and specificity of neuropsychological tests

Cognitive domains	Follow-up effect		Age effect	
	Effect on logit(Se) [CrI]	Effect on logit(Sp) [CrI]	Effect on logit(Se) [CrI]	Effect on logit(Sp) [CrI]
Paragraph delayed recall	0.071 [−0.123, 0.245]	0.070 [−0.032, 0.205]	0.145 [−0.567, 0.791]	−0.243 [−0.688, 0.399]
Word-list, free delayed recall with oriented encoding	−0.058 [−0.165, 0.014]	0.046 [−0.073, 0.151]	NA	NA
Naming	0.026 [−0.184, 0.229]	−0.094 [−0.173, −0.027]	−0.129 [−0.658, 0.354]	−0.114 [−0.399, 0.148]
Semantic fluency	0.143 [0.035, 0.261]	−0.092 [−0.242, 0.003]	−0.011 [−0.571, 0.560]	−0.064 [−0.570, 0.391]

Meta-regression results for the four cognitive domains containing five or more studies. Follow-up length and mean age were analyzed separately and both effects on Se and Sp (on the logit scale) were assessed

**Table 7:** Meta-analyses of subgroups of studies with long or short follow-up

	N	Sensitivity [CrI]	Specificity [CrI]	Follow-up (months)
Naming (short FU)	3	0.842^a^ [0.256, 1]	0.852^a^ [0.703, 0.943]	12–24
Naming (long FU)	3	0.653 [0.437, 0.825]	0.648 [0.539, 0.738]	31–36
Semantic fluency (short FU)	3	0.540 ^a^ [0.324, 0.722]	0.765 ^a^ [0.437, 0.998]	12–24
Semantic fluency (long FU)	3	0.842 [0.569, 0.966]	0.640 [0.386, 0.816]	31–36

Global estimate (median of posterior distribution) and 95% credible intervals (CrI) of sensitivity and specificity of tests assessing cognitive domains in subgroup of studies. N indicates the number of studies included in each category.

^a^For these data the second prior distribution analysis failed

### Results of the Meta-Regression

The results of the meta-regressions are presented in Table 6. There was a negative association between the length of follow-up and specificity values for the naming tests (−0.094, 95% CrI: −0.173 – -0.027: Eckerstrom et al. 2013; Gallagher et al. 2010; Ahmed et al. 2008; Galton et al. 2005; Belleville et al. 2014b; Sarazin et al. 2007). Shorter mean follow-up was associated with higher specificity values. When examining specificity in the length of follow-up subgroups (Table 7), the combined specificity value for the naming tests obtained from the three studies with the longest follow-up (31 to 36 months: Gallagher et al. 2010; Belleville et al. 2014b; Sarazin et al. 2007) was good (0.648; 95% CrI: 0.539–0.738), whereas it was very high (0.852; 95% CrI: 0.703–0.943) for the three studies with the shortest follow-up (12 to 24 months: Eckerstrom et al. 2013; Ahmed et al. 2008; Galton et al. 2005). The second prior distribution of this meta-analysis failed and thus this result should be interpreted with caution.

There was a positive association between the length of follow-up and sensitivity values for tests of semantic fluency (0.143, 95% CrI: 0.035–0.261: Gallagher et al. 2010; Galton et al. 2005; Ahmed et al. 2008; Ewers et al. 2012; Sarazin et al. 2007; Venneri et al. 2011). Longer mean follow-up was associated with higher sensitivity values. When examining sensitivity in the length of follow-up subgroups (Table 7), the three studies with the longest follow-up (31 to 36 months: Gallagher et al. 2010; Sarazin et al. 2007; Venneri et al. 2011) showed a very high sensitivity value (0.842; 95% CrI: 0.569–0.966), whereas those with the shortest follow-up (12 to 24 months) showed a low combined sensitivity of 0.540 (95% CrI: 0.324–0 .722: Galton et al. 2005; Ahmed et al. 2008; Ewers et al. 2012). There was no effect of the length of follow-up for any of the other domains. Furthermore, there was no effect of age on sensitivity or specificity values.

## Discussion

Neuropsychological assessment is central to the diagnosis of dementia and identifying individuals who may be in a prodromal phase of AD. People with MCI have a ten-fold higher risk of progressing to dementia every year than people of the same age in the general population. Thus, many people with MCI are actually in a prodromal stage of AD. However, not all patients with MCI progress to dementia and it is thus critical for clinicians to identify tools that can accurately separate those who will remain stable from those who will progress to dementia. Many longitudinal studies have published predictive accuracy values for different cognitive tests. There is a critical need for a systematic analysis of this literature because of the large number of cognitive dimensions that can be measured and because each domain can be assessed with still a larger number of neuropsychological tools.

In this systematic review, we found 28 longitudinal studies that assessed the values of neuropsychological tests to predict progression from MCI to dementia. We selected studies based on strict inclusion and exclusion criteria and hence discarded studies that contained fatal methodological flaws for a meta-analysis of predictive diagnostic test accuracy (for example, those that failed to use clinical criteria to identify progression to dementia or those for which the methodological information provided was insufficient to allow replication). Nevertheless, it was important to assess the general quality level of the remaining studies and whether the methodology could bias the data, as the studies varied somewhat. Based on the quality criteria used here (QUADAS tool and Cochrane guidelines), most studies had a relatively low risk of bias. Furthermore, the studies were relatively homogenous in their methodological approach and relied on well-accepted clinical criteria to identify their patients and outcomes. Most included studies relied on a prospective design and used Petersen’s criteria to select their participants, and all relied on the NINCDS-ARDRA to identify dementia. Yet, 10 of the 28 studies showed a high risk of bias. A few features were found to be problematic. The most frequent problem was related to the selection process, which led to the sample not being representative of the population of interest. This limitation might have a negative impact on the generalizibility of the results from these studies. Another problem was related to a failure to keep predictive tests independent from the gold standard used to identify the outcome (here, progression to dementia). This was found in five studies, and five others failed to report information regarding this criterion. This is an important methodological control because predictive accuracy can be artificially inflated when the predictors are not kept independent from the standard.

We decided to focus on measures of sensitivity and specificity and to report both independently rather than focusing mostly on overall predictive accuracy. Ideally, a test with optimal predictive accuracy should combine excellent sensitivity and specificity. However, the optimal ratio of specificity to sensitivity may also depend on the clinical and research context. For example, clinicians might favor sensitivity over specificity to detect a deadly disease that could be cured if treated. In contrast, specificity might be favored over sensitivity when a disease cannot be treated, when a diagnosis has the potential to result in stigmatization, exclusion or depression, or when treatment has important side effects. In a research context, investigators might favor a different balance between sensitivity and specificity as a function of their research question. Remarkably, our systematic review indicated that many domains and tests show an appropriate balance with very good sensitivity and specificity values.

The studies that we reviewed covered data for a total of 2365 participants who met the criteria for MCI at entry and were followed over an average of 31 months to assess whether they met the criteria for AD type dementia. In total, 916 individuals with MCI were later found to progress to dementia. This represents a progression rate of 38.7%, which is fairly consistent with the literature, considering the 31-month average follow-up (Gauthier et al. 2006). However, the progression rate across individual studies was quite variable, ranging from 6 to 39% per year.

The systematic review examined 61 cognitive tests that evaluated 22 cognitive dimensions. It identified many neuropsychological measures with very good sensitivity for predicting dementia, with some reaching more than 90%. Similarly, many neuropsychological tests revealed excellent specificity values. Five neuropsychological measures had an overall accuracy of greater than 90%. Three were episodic memory tests (Guild paragraph delayed recall, RAVLT delayed recall, and free delayed recall of names from a face-name association) and two measured visual semantics (object function recognition and the VOSP silhouette). One global test measuring different cognitive components (ACE Addenbrooke’s cognitive examination) also yielded excellent overall accuracy. Thus, although the sensitivity to specificity ratio varied for individual tests, many had an appropriate balance between the two and some showed both excellent sensitivity and specificity. This systematic review also examined the predictive value of studies that examined a combination of cognitive measures. The use of a combination of neuropsychological measures is likely to be the best approach to identify future progressors, because the sensitivity to specificity ratio varies for individual domains. Studies that have examined combined markers generally reported very high to excellent predictive accuracy with a good balance between sensitivity and specificity, particularly when they combined memory with executive or language tests.

We performed a meta-analysis of the predictive accuracy for 14 cognitive domain categories that included at least three independent studies. The meta-analysis pooled sensitivity and specificity values to obtain quantitative indicators. The meta-analysis showed that most measures of verbal memory were excellent predictors with very good (≥ 0.7) specificity and sensitivity values. In addition, predictive values from verbal memory tests were barely influenced by the testing conditions. For example, delayed recall did not predict progression better than immediate recall. Similarly, there was no major difference between cued recall and free recall and there was no added benefit from providing orientation at retrieval. This goes against the concept that measures of delayed recall or tests that orient processing at encoding are the best indicators of early AD, because they reflect hippocampal dysfunction (Dubois et al. 2007; Albert et al. 2011). The present data indicate rather that a range of verbal memory tests can be used as appropriate indicators of early AD and that the nature of the task may not profoundly influence predictive accuracy. Contrary to the general finding of high predictive value for verbal memory tasks, two testing conditions were associated with relatively low predictive accuracy: word recognition and word recall with orientation at encoding and cues at retrieval. This is consistent with the notion that AD patients suffer from impaired encoding, because being impaired on tasks that increases encoding is not a good predictor of progression.

Interestingly, some language categories were rather good predictors of future decline, particularly naming and semantic fluency. These tests implicate semantic memory and some form of executive functions, which may explain their ability to predict future decline, as both processes were proposed to be impaired early in MCI (Belleville et al. 2008; Joubert et al. 2008). The predictive accuracy of two language tests (fluency and naming) was modified as a function of the length of follow-up. This indicates that the predictive value of language categories varies with disease stage in the prodromal continuum, contrary to verbal memory, for which predictive accuracy was similar, irrespective of the prodromal stage of the patient. This finding and its implications will be discussed further below.

Several cognitive categories showed better specificity than sensitivity values. This was true for memory tasks that provide support at encoding and retrieval, for example, recognition (0.547 and 0.789 for sensitivity and specificity, respectively) or word-list cued delayed recall with oriented encoding (0.676 and 0.896 for sensitivity and specificity, respectively). The same pattern was found for some non-memory categories as well. For example shifting (sensitivity = 0.541; specificity = 0.679), working memory (sensitivity = 0.599; specificity = 0.667), and semantic knowledge (sensitivity = 0.703; specificity = 0.814) showed higher specificity than sensitivity. Thus, these tests may not be very sensitive to identify future progression, but they might be useful for identifying patients with MCI who will remain stable and thus contribute to reduce the number of false positives.

The meta-regressions examined whether age and length of follow-up determined differences in sensitivity and specificity values. Age had no effect on predictive accuracy. However, conclusions concerning age might be limited by the fact that this aspect was examined across studies and not across individuals. It is therefore possible that the search for an effect was limited by the lack of difference in the average age across studies, because disease onset is likely to be equivalent across different samples. The effect of the length of follow-up is perhaps more informative, as studies have control over this variable, which differed between studies. The predictive accuracy of naming and the semantic fluency category varied as a function of the length of follow-up. Studies with very short follow-ups might increase the likelihood of false negatives, as they do not allow sufficient time for individuals to progress to meet the dementia criteria. It is unlikely that a test would be more sensitive for a shorter than longer follow-up, as symptoms increase with progression. Thus, higher sensitivity for studies with longer follow-ups might reflect such a phenomenon. Semantic fluency followed this pattern. Fluency tasks yielded excellent sensitivity (0.842) for long follow-ups (31 to 36 months), but sensitivity markedly declined (0.540) for short follow-ups (12 to 24 months). Hence, this interaction might reflect the contribution of false negative cases to the data from studies relying on short follow-ups.

Assessing the impact of the length of follow-up is also informative for identifying very early markers. Hence, a reasonably long follow-up can be used as a proxy for how far the patient was from the diagnosis when the test was given. Determining what constitutes a reasonable follow-up is complex, but if approximately 15% of individuals with MCI progress to dementia yearly, and approximately 25% of them remain stable, irrespective of follow-up length, a three-year follow-up would allow the detection of approximately 60% of the MCI progressors, which would increase to 80% for a four-year follow-up. A task found to be predictive, irrespective of the length of follow-up, is a good candidate for an early predictor. A task found to be less predictive at a longer than shorter follow-up may be less well-suited as an early indicator of future dementia and more representative of imminent decline. This pattern was found for the naming category. Although the test showed good specificity at longer follow-ups (0.648 for follow-ups of 31 to 36 months), specificity was markedly increased, and excellent, at shorter follow-ups (0.852 for follow-ups of 12 to 24 months). Sensitivity was unaffected by the length of follow-up, suggesting that naming might fare better as a predictor at shorter rather than longer follow-up and might be better used as a marker of imminent progression rather than an early marker of the disease. This result and its interpretation needs to be confirmed by future studies and meta-analyses, because the effect was not found when examining the alternative distribution and it was based on a relatively small number of studies.

One strength of this meta-analysis was the use of the Bayesian approach, which has several advantages over other frequently used approaches. Simulation studies have shown that the Bayesian method provides better coverage probabilities for global sensitivity and specificity, particularly in the case of sparse data (Paul et al. 2010). One reason for this is that Bayesian inferences generally come with wider credibility intervals (often more realistic) than frequently used alternative methods (Warn et al. 2002). The Bayesian approach yields less biased estimations of variance and correlation parameters (Paul et al. 2010). Other frequently used approaches often experience convergence issues, which are less of an issue with the Bayesian approach (Paul et al. 2010). Finally, the Bayesian approach generally produces an approximate joint posterior distribution of all model parameters. This has the advantage of not only making it possible to test hypotheses, but also to obtain the probability that any given parameter is above or below any given threshold (Rutter and Gatsonis 2001). It also allows the easy computation of point estimates of any functions of Se and Sp, such as predictive values or likelihood ratios, along with their credibility intervals.

This study has limitations. One major limitation is that we focused on individuals meeting the criteria for MCI, which might not represent the earliest stage of AD. Thus, the follow-up periods reported in the included studies are relatively short if one considers that the disease develops over two decades prior to diagnosis. Therefore, it is unclear whether the predictive accuracy identified here is representative of earlier stages and whether it can be extended further back during the prodromal period. It is also possible that a slightly different ensemble of tasks would display different sensitivity and specificity values at an earlier period. Future studies could meta-analyze data from individuals reporting a subjective cognitive decline to assess earlier markers, as these individuals might be in a phase that precedes MCI in the disease continuum (Jessen et al. 2014). Similarly, the outcome of studies interested in pre-dementia diagnosis depends on the type of recruitment at entry and the validity of the classification scheme, for example, how subjective cognitive decline or MCI is diagnosed. Advances in the field and refinement of diagnostic criteria will certainly increase the ability to identify early markers. Another limitation is the large variability in the tasks that were tested across studies. As a result, we focused on cognitive domain categories rather than individual tasks for the meta-analyses. Although this quantitative meta-analysis provides information as to the domains that should be measured for early prediction, it does not identify specific tests. However, the systematic review included in this study identifies the predictive accuracy for a range of neuropsychological tests that map the cognitive domain categories identified in the meta-analysis. Our finding that the use of different testing conditions for memory tasks does not substantially modify predictive accuracy lends support to our approach, as it indicates that sensitivity and specificity do not vary much within broad cognitive domains. As already mentioned, the statistical analyses were limited by the small number of included studies. This does not diminish the reliability of the results, but the high between-study variability was reflected by the large CrI widths. Some statistical assumptions, for example that of normality, could not be verified due to the small number of studies. Also, the reported CrIs are not applicable to a hypothetical future study. For example, using different cut-offs would change the sensitivity and specificity values. We experienced instances of convergence failures. The Bayesian approach that we used is known to be less prone to convergence issues than other frequently used methods (Paul et al. 2010). Yet, when there are relatively few studies with sparse data, convergence can be particularly difficult to achieve because it may lead to very wide estimations of the corresponding credibility intervals. This is even more challenging for meta-regression models as they contain additional parameters. We suspect that the convergence failures were due to small amounts of data compared to the number of model parameters. Finally, sensitivity and specificity values were slightly modified in four cases after excluding studies with a high risk of bias (reduced sensitivity for three, increased specificity for one). In these cases, correct inferences for sensitivities and specificities probably fell somewhere between the original results (left-hand side of Table 5) and those excluding high-risk studies (right-hand side of Table 5).

In conclusion, the results from this meta-analysis are encouraging for those interested in the early identification of AD. They show that neuropsychological assessment, which is affordable and widely accessible, can strongly contribute to predicting dementia while individuals are still in the MCI phase. The meta-analysis revealed very good to excellent predictive accuracy for many cognitive domains, particularly those concerned with verbal memory and semantic processing. Based on the meta-analyzed data, performance on cognitive tests can predict whether MCI patients will progress to dementia at least 3 years prior to the time at which the diagnosis is made and should contribute highly to the development of early indices of AD.

## Electronic supplementary material

ESM 1 (DOCX 312 kb)

## References

[CR1] Ahmed S, Mitchell J, Arnold R, Nestor PJ, Hodges JR (2008). Predicting rapid clinical progression in amnestic mild cognitive impairment. Dementia and Geriatric Cognitive Disorders.

[CR2] Albert MS, DeKosky ST, Dickson D, Dubois B, Feldman HH, Fox NC (2011). The diagnosis of mild cognitive impairment due to Alzheimer's disease: Recommendations from the National Institute on Aging-Alzheimer's Association workgroups on diagnostic guidelines for Alzheimer's disease. Alzheimers Dement.

[CR3] Albert MS, Moss MB, Tanzi R, Jones K (2001). Preclinical prediction of AD using neuropsychological tests. Journal of the International Neuropsychological Society.

[CR4] Amieva H, Le Goff M, Millet X, Orgogozo JM, Peres K, Barberger-Gateau P (2008). Prodromal Alzheimer's disease: Successive emergence of the clinical symptoms. Annals of Neurology.

[CR5] Anchisi D, Borroni B, Franceschi M, Kerrouche N, Kalbe E, Beuthien-Beumann B (2005). Heterogeneity of brain glucose metabolism in mild cognitive impairment and clinical progression to Alzheimer disease. [Comparative Study Evaluation Studies Research Support, Non-U.S. Gov't]. Archives of Neurology.

[CR6] Arnaiz E, Jelic V, Almkvist O, Wahlund LO, Winblad B, Valind S (2001). Impaired cerebral glucose metabolism and cognitive functioning predict deterioration in mild cognitive impairment. Neuroreport.

[CR7] Association, A. P (1987). Diagnostic and statistical manual of mental disorders, ed 3, rev.

[CR8] Association, A. P (1994). Diagnostic and statistical manual of mental disorders.

[CR9] Association, A. P (2013). Diagnostic and statistical manual of mental disorders (DSM-5®).

[CR10] Babins L, Slater ME, Whitehead V, Chertkow H (2008). Can an 18-point clock-drawing scoring system predict dementia in elderly individuals with mild cognitive impairment?. Journal of Clinical & Experimental Neuropsychology: Official Journal of the International Neuropsychological Society.

[CR11] Bateman RJ, Xiong C, Benzinger TL, Fagan AM, Goate A, Fox NC (2012). Clinical and biomarker changes in dominantly inherited Alzheimer's disease. The New England Journal of Medicine.

[CR12] Belleville S, Chertkow H, Gauthier S (2007). Working memory and control of attention in persons with Alzheimer's disease and mild cognitive impairment. [Research Support, Non-U.S. Gov't]. Neuropsychology.

[CR13] Belleville S, Fouquet C, Duchesne S, Collins DL, Hudon C (2014). Detecting early preclinical Alzheimer's disease via cognition, neuropsychiatry, and neuroimaging: Qualitative review and recommendations for testing. [Research Support, Non-U.S. Gov't]. Journal of Alzheimer's Disease.

[CR14] Belleville S, Gauthier S, Lepage E, Kergoat MJ, Gilbert B (2014). Predicting decline in mild cognitive impairment: A prospective cognitive study. Neuropsychology.

[CR15] Belleville S, Sylvain-Roy S, de Boysson C, Menard MC (2008). Characterizing the memory changes in persons with mild cognitive impairment. [Research Support, Non-U.S. Gov't review]. Progress in Brain Research.

[CR16] Buchhave P, Stomrud E, Warkentin S, Blennow K, Minthon L, Hansson O (2008). Cube copying test in combination with rCBF or CSF a beta(42) predicts development of Alzheimer's disease. Dementia and Geriatric Cognitive Disorders.

[CR17] Dahabreh, I. J., Trikalinos, T. A., Lau, J., & Schmid, C. (2012). An empirical assessment of bivariate methods for meta-analysis of test accuracy. Methods Research Report. (Prepared by Tufts Evidence-based Practice Center under Contract No. 290-2007-10055-I.) AHRQ Publication No 12(13)-EHC136-EF. Rockville: Agency for Healthcare Research and Quality. November 2012. www.effectivehealthcare.ahrq.gov/reports/final/cfm. 23326899

[CR18] Defrancesco M, Marksteiner J, Deisenhammer E, Kemmler G, Djurdjevic T, Schocke M (2013). Impact of white matter lesions and cognitive deficits on conversion from mild cognitive impairment to alzheimer's disease. Journal of Alzheimer's Disease.

[CR19] Didic M, Felician O, Barbeau EJ, Mancini J, Latger-Florence C, Tramoni E (2013). Impaired visual recognition memory predicts Alzheimer's disease in amnestic mild cognitive impairment. Dementia and Geriatric Cognitive Disorders.

[CR20] Dierckx E, Engelborghs S, De Raedt R, Van Buggenhout M, De Deyn PP, Verte D (2009). Verbal cued recall as a predictor of conversion to Alzheimer's disease in mild cognitive impairment. International Journal of Geriatric Psychiatry.

[CR21] Dubois B, Feldman HH, Jacova C, Dekosky ST, Barberger-Gateau P, Cummings J (2007). Research criteria for the diagnosis of Alzheimer's disease: Revising the NINCDS-ADRDA criteria. Lancet Neurology.

[CR22] Eckerstrom C, Olsson E, Bjerke M, Malmgren H, Edman A, Wallin A (2013). A combination of neuropsychological, neuroimaging, and cerebrospinal fluid markers predicts conversion from mild cognitive impairment to dementia. Journal of Alzheimer's Disease.

[CR23] Ewers M, Walsh C, Trojanowski JQ, Shaw LM, Petersen RC, Jack CR (2012). Prediction of conversion from mild cognitive impairment to Alzheimer's disease dementia based upon biomarkers and neuropsychological test performance. [Research Support, N.I.H., Extramural Research Support, Non-U.S. Gov't]. Neurobiology of Aging.

[CR24] Flicker C, Ferris SH, Reisberg B (1991). Mild cognitive impairment in the elderly: Predictors of dementia. [Research Support, U.S. Gov't, P.H.S.]. Neurology.

[CR25] Gallagher D, Mhaolain AN, Coen R, Walsh C, Kilroy D, Belinski K (2010). Detecting prodromal Alzheimer's disease in mild cognitive impairment: Utility of the CAMCOG and other neuropsychological predictors. International Journal of Geriatric Psychiatry.

[CR26] Galton CJ, Erzinclioglu S, Sahakian BJ, Antoun N, Hodges JR (2005). A comparison of the Addenbrooke's cognitive examination (ACE), conventional neuropsychological assessment, and simple MRI-based medial temporal lobe evaluation in the early diagnosis of Alzheimer's disease. [Comparative Study Research Support, Non-U.S. Gov't]. Cognitive and Behavioral Neurology.

[CR27] Gauthier S, Patterson C, Gordon M, Soucy JP, Schubert F, Leuzy A (2011). Commentary on "recommendations from the National Institute on Aging-Alzheimer's Association workgroups on diagnostic guidelines for Alzheimer's disease." a Canadian perspective. Alzheimers Dement.

[CR28] Gauthier S, Reisberg B, Zaudig M, Petersen RC, Ritchie K, Broich K (2006). Mild cognitive impairment. Lancet.

[CR29] Griffith HR, Netson KL, Harrell LE, Zamrini EY, Brockington JC, Marson DC (2006). Amnestic mild cognitive impairment: Diagnostic outcomes and clinical prediction over a two-year time period. Journal of the International Neuropsychological Society.

[CR30] Guo, J., & Riebler, A. (2016). R package meta4diag: meta-analysis for diagnostic test studies. Trondheim: Norwegian University of Science and Technology.

[CR31] Haynes RB, Sackett DL, Guyatt GH, Tugwell P (2006). Clinical epidemiology: How to do clinical practice research.

[CR32] HIQA – IRELAND, IQWiG – GERMANY. (2015). Meta-analysis of Diagnostic Test Accuracy Studies. Eunethta. http://www.eunethta.eu/outputs/methodological-guideline-meta-analysis-diagnostic-test-accuracystudies.Accessed 14 Sep 2017.

[CR33] Irish M, Lawlor BA, Coen RF, O'Mara SM (2011). Everyday episodic memory in amnestic mild cognitive impairment: A preliminary investigation. BMC Neuroscience.

[CR34] Jessen F, Wolfsgruber S, Wiese B, Bickel H, Mosch E, Kaduszkiewicz H (2014). AD dementia risk in late MCI, in early MCI, and in subjective memory impairment. [Research Support, Non-U.S. Gov't]. Alzheimers Dement.

[CR35] Joubert S, Felician O, Barbeau EJ, Didic M, Poncet M, Ceccaldi M (2008). Patterns of semantic memory impairment in mild cognitive impairment. Behavioural Neurology.

[CR36] Kluger A, Ferris SH, Golomb J, Mittelman MS, Reisberg B (1999). Neuropsychological prediction of decline to dementia in nondemented elderly. [Research Support, U.S. Gov't, P.H.S.]. Journal of Geriatric Psychiatry and Neurology.

[CR37] Leeflang, M. G. G. (2014). Systematic reviews and meta-analyses of diagnostic test accuracy. *Clinical Microbiology and Infection* (20), 105-113. 10.1111/1469-0691.12474 24274632

[CR38] Lekeu F, Magis D, Marique P, Delbeuck X, Bechet S, Guillaume B (2010). The California verbal learning test and other standard clinical neuropsychological tests to predict conversion from mild memory impairment to dementia. Journal of Clinical and Experimental Neuropsychology.

[CR39] Liberati A, Altman DG, Tetzlaff J, Mulrow C, Gotzsche PC, Ioannidis JP (2009). The PRISMA statement for reporting systematic reviews and meta-analyses of studies that evaluate health care interventions: Explanation and elaboration. [Consensus Development Conference Guideline Research Support, Non-U.S. Gov't]. Journal of Clinical Epidemiology.

[CR40] Lischka AR, Mendelsohn M, Overend T, Forbes D (2012). A systematic review of screening tools for predicting the development of dementia. Canadian Journal on Aging.

[CR41] Macaskill, P., Gatsonis, C., Deeks, J. J., Harbord, R. M., & Takwoingi, Y. I. (2010.). Chapter 10: Analysing and presenting results. In J. J. Deeks, P. M. Bossuyt, & C. Gatsonis (Eds.), *Cochrane Handbook for Systematic Reviews of Diagnostic Test Accuracy Version 1.0. Available from:* http://Srdta.Cochrane.Org/*.* The Cochrane collaboration.

[CR42] Marcos A, Gil P, Barabash A, Rodriguez R, Encinas M, Fernandez C (2006). Neuropsychological markers of progression from mild cognitive impairment to Alzheimer's disease. [Research Support, Non-U.S. Gov't]. American Journal of Alzheimer's Disease and Other Dementias.

[CR43] Mattis S (1988). Dementia rating scale.

[CR44] McKhann G, Drachman D, Folstein M, Katzman R, Price D, Stadlan EM (1984). Clinical diagnosis of Alzheimer's disease: Report of the NINCDS-ADRDA work group under the auspices of Department of Health and Human Services Task Force on Alzheimer's disease. Neurology.

[CR45] McKhann G, Knopman DS, Chertkow H, Hyman BT, Jack CR, Kawas CH (2011). The diagnosis of dementia due to Alzheimer's disease: Recommendations from the National Institute on Aging-Alzheimer's Association workgroups on diagnostic guidelines for Alzheimer's disease. Alzheimers Dement.

[CR46] Mitchell J, Arnold R, Dawson K, Nestor PJ, Hodges JR (2009). Outcome in subgroups of mild cognitive impairment (MCI) is highly predictable using a simple algorithm. [Research Support, Non-U.S. Gov't]. Journal of Neurology.

[CR47] Ozer S, Young J, Champ C, Burke M (2016). A systematic review of the diagnostic test accuracy of brief cognitive tests to detect amnestic mild cognitive impairment. International Journal of Geriatric Psychiatry.

[CR48] Paul M, Riebler A, Bachmann LM, Rue H, L. H (2010). Bayesian bivariate meta-analysis of diagnostic test studies using integrated nested Laplace approximations. Statistics in Medicine.

[CR49] Perri R, Serra L, Carlesimo GA, Caltagirone C, Early Diagnosis Group of Italian Interdisciplinary Network on Alzheimer's, D (2007). Preclinical dementia: An Italian multicentre study on amnestic mild cognitive impairment. [Multicenter Study]. Dementia and Geriatric Cognitive Disorders.

[CR50] Perri R, Serra L, Carlesimo GA, Caltagirone C, Early Diagnosis Group of the Italian Interdisciplinary Network on Alzheimer's, D (2007). Amnestic mild cognitive impairment: Difference of memory profile in subjects who converted or did not convert to Alzheimer's disease. Neuropsychology.

[CR51] Petersen RC (2000). Mild cognitive impairment: Transition between aging and Alzheimer's disease. [Research Support, U.S. Gov't, P.H.S.]. Neurología.

[CR52] Reitsma, J., Rutjes, A. W., Whiting, P., Vlassov, V. V., Leeflang, M. M., & Deeks, J. J. (2009). Chapter 9: Assessing methodological quality. In J. J. Deeks, P. M. Bossuyt & C. Gatsonis (Eds.), The Cochrane Collaboration. http://srdta.cochrane.org/

[CR53] Reitsma JB, Glas AS, Rutjes AW, Scholten RJ, Bossuyt PM, Zwinderman AH (2005). Bivariate analysis of sensitivity and specificity produces informative summary measures in diagnostic reviews. [Review]. Journal of Clinical Epidemiology.

[CR54] Richard, E., Schmand, B. A., Eikelenboom, P., & Van Gool, W. A. (2013). MRI and cerebrospinal fluid biomarkers for predicting progression to Alzheimer's disease in patients with mild cognitive impairment: A diagnostic accuracy study. *BMJ Open, 3*(6). 10.1136/bmjopen-2012-002541. 10.1136/bmjopen-2012-002541 PMC3686215 23794572

[CR55] Robert CP (2001). Chapter 5. *The Bayesian choice: from decision-theoretic foundations to computational implementation*.

[CR56] Rue, H., Martino, S., & Chopin, N. (2009). Approximate Bayesian inference for latent Gaussian models using integrated nested Laplace approximations. *Journal of the Royal Statistical Society B* (71), 319-392.

[CR57] Rutter CM, Gatsonis CA (2001). A hierarchical regression approach to meta-analysis of diagnostic test accuracy evaluations. Statistics in Medicine.

[CR58] Sarazin M, Berr C, De Rotrou J, Fabrigoule C, Pasquier F, Legrain S (2007). Amnestic syndrome of the medial temporal type identifies prodromal AD: A longitudinal study. [Erratum appears in neurology. 2008 May 20;70(21):2016]. [Comparative Study Research Support, Non-U.S. Gov't]. Neurology.

[CR59] Saunders NL, Summers MJ (2010). Attention and working memory deficits in mild cognitive impairment. Journal of Clinical and Experimental Neuropsychology.

[CR60] Spiegelhalter DJ, Myles JP, Jones DR, Abrams KR (1999). An introduction to Bayesian methods in health technology assessment. BMJ.

[CR61] Summers MJ, Saunders NL (2012). Neuropsychological measures predict decline to Alzheimer's dementia from mild cognitive impairment. Neuropsychology.

[CR62] Tabert MH, Manly JJ, Liu X, Pelton GH, Rosenblum S, Jacobs M (2006). Neuropsychological prediction of conversion to alzheimer disease in patients with mild cognitive impairment. Archives of General Psychiatry.

[CR63] Tierney MC, Szalai JP, Snow WG, Fisher RH, Nores A, Nadon G (1996). Prediction of probable Alzheimer's disease in memory-impaired patients: A prospective longitudinal study. [Research Support, Non-U.S. Gov't]. Neurology.

[CR64] Tsoi KK, Chan JY, Hirai HW, Wong SY, Kwok TC (2015). Cognitive tests to detect dementia: A systematic review and meta-analysis. JAMA Internal Medicine.

[CR65] Venneri A, Gorgoglione G, Toraci C, Nocetti L, Panzetti P, Nichelli P (2011). Combining neuropsychological and structural neuroimaging indicators of conversion to alzheimer's disease in amnestic mild cognitive impairment. Current Alzheimer Research.

[CR66] Visser PJ, Verhey FRJ, Ponds R, Jolles J (2001). Diagnosis of preclinical Alzheimer's disease in a clinical setting. [Article]. International Psychogeriatrics.

[CR67] Whiting, P., Rutjes, A. W., Reitsma, J. B., Bossuyt, P. M., & Kleijnen, J. (2003). The development of QUADAS: A tool for the quality assessment of studies of diagnostic accuracy included in systematic reviews. [Evaluation Studies Research Support, Non-U.S. Gov't]. *BMC Medical Research Methodology, 3*, 25, 10.1186/1471-2288-3-25. 10.1186/1471-2288-3-25 PMC305345 14606960

[CR68] Warn DE, Thompson SG, Spiegelhalter DJ (2002). Bayesian random effects meta-analysis of trials with binary outcomes: Methods for the absolute risk difference and relative risk scales. Statistics in Medicine.

[CR69] Whiting PF, Rutjes AW, Westwood ME, Mallett S, Deeks JJ, Reitsma JB (2011). QUADAS-2: A revised tool for the quality assessment of diagnostic accuracy studies. [Research Support, Non-U.S. Gov't]. Annals of Internal Medicine.

