# Supplementary material for: Neuropsychological Measures that Predict Progression from Mild Cognitive Impairment to Alzheimer's type dementia in Older Adults: a Systematic Review and Meta-Analysis
Source: Neuropsychol Rev. 2017 Oct 10;27(4):328–53. doi: 10.1007/s11065-017-9361-5 (PMC5754432; doi:10.1007/s11065-017-9361-5)
Supplement: Supplementary file 1 — (DOCX 312 kb) [file 11065_2017_9361_MOESM1_ESM.docx]

***SUPPLEMENTARY MATERIAL***

**APPENDIX A**

**Electronic search strategy for the MEDLINE database**

**Concept 1: Elderly people**

aging/ or aged/ or "aged, 80 and over"/ or middle aged/ or (((aged* or old*) and (people or person or adult or man or men or woman or women or patient*)) or (elder* or ageing or aging or senior or older)).m_titl. or (((aged* or old*) and (people or person or adult or man or men or woman or women or patient*)) or (elder* or aging or ageing or senior or older)).ab.

- **#1 (4 204 911 refs)**

**Concept 2: MCI**

"Mild cognitive impairment"/ or Memory disorders/ or ("memory disorder*" or "memory impairment*" or "memory loss*" or prodromal Alzheimer disease or "mild cognitive impairment*" or preclinical Alzheimer disease or predementia or "memory deficit*" or MCI or "cognitive deficit*" or "cognitive decline*" or "cognitive impairment*" or early stage dementia or "mild memory problem*" or "mild memory difficult*" or questionable dementia or cognitive deterioration).m_titl. or ("memory disorder*" or "memory impairment*" or "memory loss*" or prodromal Alzheimer disease or "mild cognitive impairment*" or preclinical Alzheimer disease or predementia or "memory deficit*" or MCI or "cognitive deficit*" or "cognitive decline*" or "cognitive impairment*" or early stage dementia or "mild memory problem*" or "mild memory difficult*" or questionable dementia or cognitive deterioration).ab.

- **#2 (66 368 refs)**

**Concept 3: Index tests**

cognition/ or memory/ or psychological tests/ or neuropsychological tests/ or activities of daily living/ or (neuropsychological measure* or neuropsychological test* or cognitive test* or cognitive assessment* or cognitive evaluation or learning test* or neuropsychological assessment* or memory assessment* or memory test* or neuropsychological follow-up or functional activities questionnaires or functional status or activities of daily living or functional activity questionnaire or functional performances or daily functioning or ((cogniti* or memory or neuropsycholog*) and (screening or tests battery))).m_titl. or (neuropsychological measure* or neuropsychological test* or cognitive test* or cognitive assessment* or cognitive evaluation or learning test* or neuropsychological assessment* or memory assessment* or memory test* or neuropsychological follow-up or functional activities questionnaires or functional status or activities of daily living or functional activity questionnaire or functional performances or daily functioning or ((cogniti* or memory or neuropsycholog*) and (screening or tests battery))).ab.

- **# 3 (257 045 refs)**

**Concept 4: Prediction**

"disease progression"/ or "early diagnosis"/ or "predictive value of tests"/ or prognosis/ or "sensitivity and specificity"/ or "severity of illness index"/ or (predict* or prognos* or early detect* or conversion or convert* or progression or early diagnos* or progressor or predictive accuracy or progres* or (sensitivity and specificity)).m_titl. or (predict* or prognos* or early detect* or conversion or convert* or progression or early diagnos* or progressor or predictive accuracy or progres* or (sensitivity and specificity)).ab.

- **#4 (2 428 984 refs)**

**Concept 5: Alzheimer's Disease**

Alzheimer Disease/co, di, ep, et, px or cognition disorders/co, di, ep, et, px or memory disorders/co, di, ep, et, px or dementia/co, di, ep, et, px or (memory disorder* or cognitive disorder or memory loss* or prodromal Alzheimer disease or cognitive decline or cognitive deterioration or early-stage dementia or Alzheimer's disease or Alzheimer disease or dementia).m_titl. or (memory disorder* or cognitive disorder* or memory loss* or prodromal Alzheimer disease or cognitive decline or cognitive deterioration or early-stage dementia or Alzheimer's disease or Alzheimer disease or dementia).ab.

- **#5 (157 972 refs)**

**Concept 6: Longitudinal studies**

Cohort studies/ or follow up studies/ or prospective studies/ or longitudinal studies/ or retrospective studies/ or (cohort stud* or prospective stud* or follow up stud* or longitudinal stud* or retrospective stud* or prospective cohort stud* or community stud* or population-based stud*).m_titl. or (cohort stud* or prospective stud* or follow up stud* or longitudinal stud* or retrospective stud* or prospective cohort stud* or community stud* or population-based stud*).ab.

**#6 (1 382 195 refs)**

**Combined search:**

**#1 AND #2 AND #3 AND #4 AND #5 AND #6 = #7 = 2637 references**

**7. LIMITES & EXCLUSION**

**-ENGLISH AND FRENCH:** limit #7 to (english or french)

- **#8 = 2570 references**

**- SPECIFIC POPULATION:** #9

hiv/ or hiv-1/ or hiv-2/ or basal ganglia diseases/ or exp basal ganglia cerebrovascular disease/ or chorea gravidarum/ or dystonia musculorum deformans/ or hepatolenticular degeneration/ or huntington disease/ or meige syndrome/ or exp multiple system atrophy/ or neuroleptic malignant syndrome/ or pantothenate kinase-associated neurodegeneration/ or exp parkinsonian disorders/ or supranuclear palsy, progressive/ or tourette syndrome/ or exp Diabetes Mellitus/ or exp Multiple Sclerosis/ or exp Schizophrenia/ or Respiratory Distress Syndrome, Adult/

- **Final result with exclusion: #8 not #9 = #10**

**= 2259 references**

- **Final results without duplicates: #10 - 46 duplicates**

**= 2213 references**

**Table S1**

**Data collected from the studies**

| **General description of the study** | |
| --- | --- |
| Authors | |
| Year of publication | |
| Country | |
| Study design | |
| Data source | |
| **Participants** |  |
| Recruitment period | |
| Total population size | |
| Sample size at baseline | |
| Recruitment rate | |
| Loss to follow-up | |
| Mean/Median age | |
| % of female participants | |
| Inclusion/exclusion criteria | |
| Criteria for MCI at baseline | |
| Criteria used to determine AD at follow-up | |
| **Methodological features** | |
| Description of standard tests and predictive tests | |
| Independence between the tests used for prediction and the tests used for diagnosis | |
| **Results** |  |
| Number of participants who converted to AD | |
| Number of participants who remained stable | |
| Conversion rate | |
| Demographic and clinical characteristics of participants who converted vs. remained stable (age, sex, education, MMSE, score at baseline on) | |
| Mean score at baseline of participants who converted vs. remained stable | |
| Predictive data: Sensitivity, specificity, overall accuracy, TN, TP, FP, FN of each studies test | |
| Cut-off of neuropsychological tests used for analysis | |
| **Analysis and analytic procedure** | |
| Type of statistical analysis | |

**Table S2**

***Adaptation of the QUADAS 2 tool to assess risk of bias in individual studies***

| **Domain** | **Bias or variation** | **Signalling questions** | **Answers indicating potential high risk of bias, imprecision, or concerns about applicability** |
| --- | --- | --- | --- |
| **Patient selection / Population** | | | |
|  | Variation in participants selection | Was the spectrum of participants representative of the participants/patients who will receive the test in practice? | Convenience sample |
|  | Disease prevalence variation/bias | Could the selection of patients have introduced bias (inclusion/exclusion criteria, recruitment sites)? | Inappropriate exclusion |
|  | Disease severity variation | Is the studied population well defined and relevant for our study? | No clear objective criteria for patients classification/selection |
| **Study design** | | | |
|  | Variation specific to prognostic studies | Is the design of the study adapted to the assessment of the predictive value of tests? | Retrospective study |
|  | Incorporation bias | Was there independence between diagnostic (standard test) and predictive batteries (index test)? | Some tests used for diagnostic were included in the predictive battery |
|  | Detection bias | Test review & diagnostic review variation: Were the evaluators blind to diagnostic or predictive tests? | The diagnostic tests results were interpreted with knowledge of the predictive tests results (in case of retrospective study) |
| **Flow and Follow-up** | | | |
|  | Variation specific to prognostic studies | Was the follow-up sufficiently long? | 12-month follow-up |
|  | Handling of un-interpretable or missing results bias | Was the loss to follow-up explained? | Reasons for loss to follow-up were not explained |
|  |  | Could the loss to follow-up rate be linked to the testing procedure (such as a too important/weighty predictive testing battery)? | High loss to follow-up rate caused by the testing procedure (>30%/year) |
| **Reference standard / Outcome** | | | |
|  | Use of an inappropriate reference standard | Was the outcome objective (AD dementia), clearly defined with well-recognized criteria? | Criteria not clearly defined |
|  | Partial verification bias | Did the whole sample receive verification using reference standard of diagnosis? | Several methods were used for the diagnostic or a sample of participants did not receive the diagnostic procedure at the end of follow-up |
|  |  |  |  |
| **Index tests/ Prognostic variable** | | | |
|  | Test execution variation | Was the index test (or predictive testing battery) well described (sufficient details to permit replication of the tests)? | Absence of methodological details allowing replication |
| **Analysis** | | | |
|  | Variation specific to prognostic study | Did the data analysis allow for the identification of the optimal subset of predictors that are uniquely associated with the outcome, and thus yield a model of AD prediction with no redundant predictors? | A priori selection of one or several tests for predictive accuracy analysis. |
|  | Arbitrary choice of threshold value (variation) | If a threshold was used, was it pre-specified? | Index test threshold was derived from the same data set in which test performance was evaluated |

.

**Table S3**

**Results of individual studies, grouped by cognitive domains**

|  | **N** | **TP** | **FP** | **FN** | **TN** | **SE** | **SP** | **ACC** | **Cut-off** |
| --- | --- | --- | --- | --- | --- | --- | --- | --- | --- |
| **Verbal episodic memory** |  |  |  |  |  |  |  |  |  |
| **Verbal immediate recall**** | **7** |  |  |  |  |  |  |  |  |
| Logical memory, immediate recall (Galton et al. 2005)* |  | 10 | 4 | 1 | 14 | 0.91 | 0.78 | 0.83 | n.r. |
| Prose recall, immediate recall (Perri et al. 2007b)* |  | 28 | 27 | 51 | 84 | 0.35 | 0.76 | 0.59 | n.r. |
| FCSRT, free recall (Didic et al. 2013)* |  | 12 | 2 | 3 | 9 | 0.80 | 0.82 | 0.81 | 17 |
| FCSRT, free recall (Sarazin et al. 2007)*^1^ |  | 42 | 13 | 17 | 145 | 0.71 | 0.92 | 0.86 | 17 |
| RAVLT, total recall (Richard et al. 2013)*^2^ |  | 71 | 52 | 10 | 48 | 0.88 | 0.48 | 0.66 | 34.59 |
| Unrelated word list, immediate recall (Perri et al. 2007b) |  | 28 | 24 | 51 | 87 | 0.35 | 0.78 | 0.61 | n.r. |
| Related word list, immediate recall (Perri et al. 2007b) |  | 40 | 34 | 39 | 77 | 0.51 | 0.69 | 0.62 | n.r. |
| **World-list cued immediate recall with oriented encoding** | **2** |  |  |  |  |  |  |  |  |
| FCSRT, total recall (Didic et al. 2013) |  | 10 | 1 | 5 | 10 | 0.67 | 0.91 | 0.77 | 37 |
| FCSRT, total recall (Sarazin et al. 2007)^1^ |  | 47 | 16 | 12 | 142 | 0.80 | 0.90 | 0.87 | 40 |
| **Paragrap delayed recall**** | **5** |  |  |  |  |  |  |  |  |
| Logical memory, delayed recall (Didic et al. 2013)* |  | 11 | 0 | 4 | 11 | 0.73 | 1 | 0.85 | 6 |
| Logical memory, delayed recall (Galton et al. 2005)* |  | 10 | 4 | 1 | 14 | 0.91 | 0.78 | 0.83 | n.r. |
| Macro-TEXT, delayed recall (Belleville et al. 2014b)* |  | 31 | 5 | 27 | 27 | 0.53 | 0.84 | 0.64 | 13/24 |
| Guild Paragraph, delayed recall (Kluger et al. 1999)* |  | 45 | 4 | 2 | 20 | 0.96 | 0.83 | 0.92 | n.r. |
| Prose recall, delayed recall (Perri et al. 2007b)* |  | 47 | 40 | 32 | 71 | 0.6 | 0.64 | 0.62 | n.r. |
| **Word-list free delayed recall with non oriented encoding**** | **5** |  |  |  |  |  |  |  |  |
| RAVLT, delayed Recall (Eckerstrom et al. 2013)* ^3^ |  | 12 | 1 | 1 | 20 | 0.92 | 0.94 | 0.94 | n.r. |
| RAVLT, delayed recall (Ewers et al. 2012)* |  | 45 | 36 | 13 | 36 | 0.78 | 0.49 | 0.62 | n.r. |
| RAVLT, delayed recall (Visser et al. 2001)* |  | 17 | 3 | 6 | 41 | 0.74 | 0.93 | 0.87 | n.r. |
| Unrelated word list_delayed recall (Perri et al. 2007b)* |  | 50 | 32 | 29 | 79 | 0.63 | 0.71 | 0.68 | n.r. |
| Related word list_delayed recall (Perri et al. 2007b)* |  | 61 | 46 | 18 | 65 | 0.77 | 0.59 | 0.66 | n.r. |
| **Word-list free delayed recall with oriented encoding**** | **5** |  |  |  |  |  |  |  |  |
| CVLT, long delay free recall (Anchisi et al. 2005)* |  | 13 | 14 | 1 | 20 | 0.93 | 0.59 | 0.69 | 7 |
| Delayed Word Recall, extended free recall (Gallagher et al. 2010)* |  | 49 | 10 | 15 | 32 | 0.77 | 0.76 | 0.76 | 3 |
| FCSRT, delayed free recall  (Sarazin et al. 2007)* ^1^ |  | 45 | 15 | 14 | 143 | 0.76 | 0.91 | 0.87 | 6 |
| RL/RI 16, free delayed recall (Belleville et al. 2014b)* |  | 38 | 8 | 15 | 24 | 0.72 | 0.75 | 0.73 | 6 |
| Face-name association task, free delayed recall of names (Irish et al. 2011)* |  | 6 | 1 | 0 | 8 | 1 | 0.86 | 0.92 | 3 |
| **Word-list cued delayed recall with oriented encoding **** | **3** |  |  |  |  |  |  |  |  |
| FCSRT, total delayed recall (Sarazin et al. 2007)* ^1^ |  | 41 | 18 | 18 | 140 | 0.69 | 0.89 | 0.83 | 14 |
| FCSRT, total delayed recall (Didic et al. 2013)* |  | 9 | 1 | 6 | 10 | 0.60 | 0.91 | 0.73 | 12 |
| MISplus total delayed recall free&cued (Dierckx et al. 2009)* |  | 5 | 2 | 2 | 22 | 0.71 | 0.92 | 0.87 | 2 |
| **Word-list recognition**** | **3** |  |  |  |  |  |  |  |  |
| Warrington's recognition memory tests, Short recognition memory test for words (Galton et al. 2005)* |  | 7 | 2 | 4 | 16 | 0.64 | 0.89 | 0.79 | n.r. |
| RAVLT, delayed recognition (Ewers et al. 2012)* |  | 29 | 25 | 29 | 47 | 0.50 | 0.65 | 0.58 | n.r. |
| Delayed Word Recall, extended recognition (Gallagher et al. 2010)* |  | 36 | 6 | 28 | 36 | 0.57 | 0.85 | 0.68 | 10 |
| **Paragraph recognition** | **1** |  |  |  |  |  |  |  |  |
| Logical memory WMS-III Recognition (Didic et al. 2013) |  | 5 | 0 | 6 | 9 | 0.45 | 1 | 0.70 | 18 |
| **Visual episodic memory** |  |  |  |  |  |  |  |  |  |
| **Immediate recall** | **2** |  |  |  |  |  |  |  |  |
| Benton visual retention test (Sarazin et al. 2007)^1^ |  | 25 | 36 | 34 | 122 | 0.42 | 0.77 | 0.68 | 11 |
| Rey's figure, immediate recall (Perri et al. 2007b) |  | 14 | 9 | 65 | 102 | 0.18 | 0.92 | 0.61 | n.r. |
| **Delayed recall**** | **4** |  |  |  |  |  |  |  |  |
| Rey's figure, delayed recall (Didic et al. 2013)* |  | 12 | 4 | 2 | 7 | 0.86 | 0.64 | 0.76 | 10 |
| Visual Reproduction, percent retention (Griffith et al. 2006)* |  | 10 | 3 | 3 | 33 | 0.77 | 0.91 | 0.88 | 26 |
| Rey's figure, delayed recall (Lekeu et al. 2010)* |  | 13 | 4 | 4 | 13 | 0.76 | 0.76 | 0.76 | 11.6 |
| Rey's figure, delayed recall (Perri et al. 2007b)* |  | 28 | 13 | 51 | 98 | 0.35 | 0.88 | 0.66 | n.r. |
| **Visual recognition** | **5** |  |  |  |  |  |  |  |  |
| DMS48, delayed recognition (Didic et al. 2013) |  | 12 | 2 | 3 | 9 | 0.80 | 0.82 | 0.81 | 91 |
| Doors, total score (Galton et al. 2005) |  | 7 | 5 | 4 | 13 | 0.64 | 0.72 | 0.69 | n.r. |
| DSM48, immediate recognition (Didic et al. 2013) |  | 12 | 1 | 3 | 10 | 0.80 | 0.91 | 0.85 | 89 |
| Warrington's recognition memory tests, Short recognition memory test for faces (Galton et al. 2005) |  | 6 | 2 | 5 | 16 | 0.55 | 0.89 | 0.76 | n.r. |
| Face recognition WMS-III, immediate scaled score (Didic et al. 2013) |  | 14 | 6 | 1 | 5 | 0.93 | 0.45 | 0.73 | 12 |
| **Associative memory** | **5** |  |  |  |  |  |  |  |  |
| Paired Associate Learning (Venneri et al. 2011) |  | 11 | 14 | 0 | 0 | 1.00 | 0.00 | 0.44 | n.r. |
| CANTAB paired associate learning_short version (Ahmed et al. 2008) |  | 7 | 5 | 0 | 6 | 1.00 | 0.55 | 0.72 | 14 |
| Visual Association test total (Dierckx et al. 2009) |  | 3 | 1 | 4 | 23 | 0.43 | 0.96 | 0.84 | 1/6 |
| Famous buildings, Associative Learning Battery, errors (Ahmed et al. 2008) |  | 6 | 6 | 1 | 5 | 0.86 | 0.46 | 0.61 | 10 |
| Patterns, Associative Learning Battery, errors (Ahmed et al. 2008) |  | 7 | 6 | 0 | 5 | 1.00 | 0.46 | 0.67 | 18 |
| **Language** |  |  |  |  |  |  |  |  |  |
| **Naming tests**** | **7** |  |  |  |  |  |  |  |  |
| Boston naming test; 60 items (Eckerstrom et al. 2013)* ^3^ |  | 13 | 5 | 0 | 16 | 0.99 | 0.74 | 0.85 | n.r. |
| Boston naming test; 30 items (Gallagher et al. 2010)* |  | 52 | 18 | 12 | 24 | 0.81 | 0.58 | 0.72 | 24 |
| Graded naming test (Ahmed et al. 2008)* |  | 3 | 1 | 4 | 10 | 0.40 | 0.91 | 0.72 | 14 |
| Picture naming from the Cambridge Semantic Battery ; Naming _64 (Galton et al. 2005)* |  | 8 | 2 | 3 | 16 | 0.73 | 0.89 | 0.83 | n.r. |
| D.O.80 (Belleville et al. 2014b)* |  | 33 | 11 | 26 | 22 | 0.56 | 0.67 | 0.60 | 78 |
| Deno 100  (Sarazin et al. 2007) ***^1^ |  | 33 | 51 | 26 | 107 | 0.56 | 0.68 | 0.65 | 89 |
| Graded naming test (Galton et al. 2005) |  | 9 | 4 | 2 | 14 | 0.82 | 0.78 | 0.79 | n.r. |
| **Tests of Semantic knowledge**** | **5** |  |  |  |  |  |  |  |  |
| Knowledge of public events_total recall (Didic et al. 2013)* |  | 10 | 2 | 3 | 9 | 0.77 | 0.82 | 0.79 | 26 |
| Similarities WAIS-R (Sarazin et al. 2007) *^1^ |  | 29 | 44 | 30 | 114 | 0.49 | 0.72 | 0.66 | 11 |
| Object function recognition (Flicker et al. 1991)* |  | 20 | 0 | 3 | 9 | 0.86 | 1.00 | 0.90 | n.r. |
| Object identification (Flicker et al. 1991) |  | 13 | 0 | 10 | 9 | 0.57 | 1.00 | 0.69 | n.r. |
| Knowledge of public events_free recall (Didic et al. 2013) |  | 10 | 3 | 3 | 8 | 0.77 | 0.73 | 0.75 | 8 |
| **Phonemic fluency** | **2** |  |  |  |  |  |  |  |  |
| Letter fluency; CAMCOG (Gallagher et al. 2010) |  | 33 | 15 | 31 | 27 | 0.52 | 0.64 | 0.57 | 28 |
| Letter fluency_ S (Sarazin et al. 2007) ^1^ |  | 34 | 69 | 25 | 89 | 0.58 | 0.56 | 0.57 | 17 |
| **Semantic fluency**** | **6** |  |  |  |  |  |  |  |  |
| Category fluency; animals, vegetables & fruits (Gallagher et al. 2010)* |  | 60 | 22 | 4 | 20 | 0.94 | 0.48 | 0.75 | 36 |
| Category fluency; animals, fruits, and household (Galton et al. 2005)* |  | 8 | 6 | 3 | 12 | 0.73 | 0.67 | 0.69 | n.r. |
| Category fluency; Animals (Ahmed et al. 2008)* |  | 2 | 0 | 5 | 11 | 0.29 | 1.00 | 0.72 | 11 |
| Category fluency; vegetables (Ewers et al. 2012)* |  | 32 | 25 | 26 | 47 | 0.55 | 0.65 | 0.61 | n.r. |
| Category fluency; fruits (Sarazin et al. 2007) *^1^ |  | 33 | 28 | 26 | 130 | 0.56 | 0.82 | 0.75 | 13 |
| Category fluency (Venneri et al. 2011)* |  | 10 | 7 | 1 | 7 | 0.91 | 0.50 | 0.68 | n.r. |
| **Executive functions and working memory** |  |  |  |  |  |  |  |  |  |
| **Executive/Switching functions**** | **5** |  |  |  |  |  |  |  |  |
| Trail making test, Part B time (Ahmed et al. 2008)* |  | 3 | 5 | 3 | 6 | 0.50 | 0.55 | 0.50 | 128 |
| Trail making test, Part B time (Sarazin et al. 2007)*^1^ |  | 37 | 52 | 22 | 106 | 0.63 | 0.67 | 0.66 | 138 |
| Trail making test, Part B time; 3Year FU (Ewers et al. 2012)* |  | 4 | 3 | 7 | 12 | 0.36 | 0.80 | 0.62 | n.r. |
| Digit symbol test WAIS-R (Sarazin et al. 2007)^1^ |  | 22 | 45 | 37 | 113 | 0.37 | 0.72 | 0.62 | 10 |
| Stroop test; time inhibition condition (Sarazin et al. 2007)^1^ |  | 31 | 66 | 28 | 92 | 0.53 | 0.58 | 0.57 | 59 |
| **Working memory tests**** | **4** |  |  |  |  |  |  |  |  |
| Digit span, total score (Ewers et al. 2012)* |  | 30 | 24 | 28 | 48 | 0.51 | 0.66 | 0.60 | n.r. |
| Serial Digit learning Test (Sarazin et al. 2007)* ^1^ |  | 34 | 51 | 25 | 107 | 0.58 | 0.68 | 0.65 | 80 |
| Alpha span, items alpha recalled (Belleville et al. 2014b)* |  | 40 | 12 | 17 | 21 | 0.70 | 0.64 | 0.68 | 58 |
| Double task baddeley (Sarazin et al. 2007) ^1^ |  | 30 | 69 | 29 | 89 | 0.51 | 0.56 | 0.55 | 94 |
| **Visuo-constructive functions** |  |  |  |  |  |  |  |  |  |
| **Visuo-spatial tests**** | **5** |  |  |  |  |  |  |  |  |
| CAMCOG, Perception score (Gallagher et al. 2010)* |  | 24 | 8 | 40 | 34 | 0.37 | 0.80 | 0.55 | 9 |
| CAMCOG, Perception score (Marcos et al. 2006)* |  | 32 | 17 | 6 | 27 | 0.84 | 0.61 | 0.72 | 7.5 |
| BORB object decision (Belleville et al. 2014b)* |  | 29 | 10 | 25 | 23 | 0.54 | 0.70 | 0.60 | 29 |
| VOSP Silhouettes (Eckerstrom et al. 2013)*^3^ |  | 12 | 2 | 1 | 19 | 0.92 | 0.92 | 0.91 | n.r. |
| BORB Line judgment (Belleville et al. 2014b) |  | 34 | 15 | 20 | 18 | 0.63 | 0.55 | 0.60 | n.r. |
| **Visuo-constructive tasks**** | **4** |  |  |  |  |  |  |  |  |
| Clock drawing test 18 point scoring system (Babins et al. 2008)* |  | 32 | 21 | 9 | 20 | 0.78 | 0.49 | 0.63 | 15 |
| Cube Copying, total Score (Buchhave et al. 2008)* |  | 34 | 24 | 29 | 60 | 0.54 | 0.71 | 0.64 | 13 |
| Block design WAIS-R (Arnaiz et al. 2001)* |  | 4 | 2 | 5 | 9 | 0.44 | 0.82 | 0.65 | n.r. |
| Raven's progressive matrices_ PM47 (Venneri et al. 2011)* |  | 8 | 6 | 3 | 8 | 0.73 | 0.57 | 0.64 | n.r. |
| **Brief and Global measures** |  |  |  |  |  |  |  |  |  |
| **Global measures**** | **6** |  |  |  |  |  |  |  |  |
| ACE Addenbrooke’s Cognitive Examination (Ahmed et al. 2008)* |  | 7 | 5 | 0 | 6 | 1.00 | 0.55 | 0.72 | 88 |
| ACE Addenbrooke’s Cognitive Examination (Galton et al. 2005)* |  | 8 | 0 | 3 | 18 | 0.73 | 1.00 | 0.90 | n.r. |
| CAMCOG, total score (Gallagher et al. 2010)* |  | 51 | 13 | 13 | 29 | 0.80 | 0.68 | 0.75 | 86 |
| CAMCOG, total score (Marcos et al. 2006)* |  | 35 | 14 | 3 | 30 | 0.92 | 0.68 | 0.79 | 79.5 |
| ADAS-Cog_total score (Galton et al. 2005) |  | 7 | 2 | 4 | 16 | 0.64 | 0.89 | 0.79 | n.r. |
| MMSE (Marcos et al. 2006) |  | 32 | 18 | 6 | 26 | 0.84 | 0.59 | 0.71 | 28.5 |
| **Orientation** | **2** |  |  |  |  |  |  |  |  |
| CAMCOG_ Orientation score (Gallagher et al. 2010) |  | 24 | 5 | 40 | 38 | 0.38 | 0.89 | 0.58 | 9 |
| CAMCOG_ Orientation score (Marcos et al. 2006) |  | 33 | 21 | 5 | 23 | 0.87 | 0.52 | 0.68 | 9.5 |
|  |  |  |  |  |  |  |  |  |  |

N = number of study results; TP = number of true positive; FP = number of false positive; FN = number of false negative; TN = number of true negative; SE = sensitivity; SP = specificity; ACC = overall accuracy; n.r. = not reported; ** Cognitive domains included in a meta-analysis; *data included in a meta-analysis. ^1^ Age corrected data; ^2^Age, gender and education corrected data; ^3^Age and education corrected data.

**Table S4**

**Results of individual studies presenting predictive values of a combination of neuropsychological test**

| **Cognitive domains** | **TP** | **FP** | **FN** | **TN** | **SE** | **SP** | **ACC** | **Covariates** |
| --- | --- | --- | --- | --- | --- | --- | --- | --- |
| **Episodic memory + verbal recognition** | | |  |  |  |  |  |  |
| DWR_free recall + DWR recognition (Gallagher et al. 2010) | 28 | 4 | 36 | 38 | 0.44 | 0.90 | 0.62 |  |
| **Episodic memory + visual memory recognition** | | |  |  |  |  |  |  |
| Logical Memory WMS-III delayed recall + DMS48 immediate recognition (Didic et al. 2013) | 15 | 0 | 0 | 11 | 1 | 1 | 1 |  |
| **Episodic memory + Executive functions** | | |  |  |  |  |  |  |
| Figures WMS immediate recall + Trail making test, Part B Time + Self Ordering Test total score (Albert et al. 2001) | 17 | 15 | 6 | 74 | 0.74 | 0.83 | 0.80 | Age, gender education |
| Dementia Rating Scale_initiation/perserveration (36 cut-off) + Visual Reproduction_percent retention (Griffith et al. 2006) | 10 | 3 | 3 | 33 | 0.77 | 0.92 | 0.88 |  |
| SRT_total immediate recall + Digit symbol test WAIS-R (Tabert et al. 2006) | 26 | 8 | 8 | 72 | 0.76 | 0.90 | 0.86 | Age |
| Combinaison RAVLT_delayed recall + Mental control WMS (Tierney et al. 1996) | 22 | 6 | 7 | 88 | 0.76 | 0.94 | 0.89 | Age and education |
| **Episodic memory + Global measures** | | |  |  |  |  |  |  |
| Rey's Figure delayed recall + Unrelated Word list delayed recall + Recognition d' + CDR mean sum boxes (Perri et al. 2007b) | 44 | 14 | 23 | 87 | 0.66 | 0.86 | 0.78 | Age |
| **Episodic memory + brief measures** | | |  |  |  |  |  |  |
| CERAD word list recall + MMSE orientation (Defrancesco et al. 2013) | 23 | 3 | 8 | 26 | 0.74 | 0.90 | 0.82 | Age |
| **Episodic memory + language** |  |  |  |  |  |  |  |  |
| DWR_free recall + category fluency (Gallagher et al. 2010) | 54 | 9 | 10 | 33 | 0.84 | 0.79 | 0.82 |  |
| **Episodic memory + global measures + visual perception** | | |  |  |  |  |  |  |
| Combination CAMCOG global score + camcog memory + camcog perception (Marcos et al. 2006) | 35 | 12 | 3 | 32 | 0.92 | 0.73 | 0.82 |  |
| **Episodic memory + language + visual perception + executive functions** | | |  |  |  |  |  |  |
| Macro-text, delayed recall + Free recall of words +D.O.80 + BORB line judgment + BORB object decision +alpha span (Belleville et al. 2014b) | 44 | 4 | 6 | 27 | 0.88 | 0.87 | 0.88 | Age |
| **Associative memory + Global measures** | | |  |  |  |  |  |  |
| Addenbrooke’s Cognitive Examination + CANTAB paired associate learning (Ahmed et al. 2008) | 7 | 2 | 0 | 9 | 1 | 0.82 | 0.89 |  |
| Addenbrooke’s Cognitive Examination and/or CANTAB paired associate learning (Mitchell et al. 2009) | 33 | 27 | 2 | 18 | 0.94 | 0.40 | 0.64 |  |
| **Associative memory + language** |  |  |  |  |  |  |  |  |
| Paired associates learning + category fluency (Venneri et al. 2011) | 10 | 7 | 1 | 7 | 0.91 | 0.50 | 0.68 |  |
| **Associative memory + visuo-construction** | | |  |  |  |  |  |  |
| Paired associates learning + Raven's matrices (Venneri et al. 2011) | 8 | 6 | 3 | 8 | 0.73 | 0.57 | 0.64 |  |
| **Associative memory + visuo-construction + language** | | |  |  |  |  |  |  |
| Paired associates learning + Raven's matrices + category fluency (Venneri et al. 2011) | 7 | 7 | 4 | 7 | 0.64 | 0.50 | 0.56 |  |

N = number of study results; TP = number of true positive; FP = number of false positive; FN= number of false negative; TN = number of true negative; SE = sensitivity; SP = specificity; ACC= overall accuracy
